# Patient-tailored adoptive immunotherapy with EBV-specific T cells from related and unrelated donors

**DOI:** 10.1172/JCI163548

**Published:** 2023-06-15

**Authors:** Agnes Bonifacius, Britta Lamottke, Sabine Tischer-Zimmermann, Rebecca Schultze-Florey, Lilia Goudeva, Hans-Gert Heuft, Lubomir Arseniev, Rita Beier, Gernot Beutel, Gunnar Cario, Birgit Fröhlich, Johann Greil, Leo Hansmann, Justin Hasenkamp, Michaela Höfs, Patrick Hundsdoerfer, Edgar Jost, Kinan Kafa, Oliver Kriege, Nicolaus Kröger, Stephan Mathas, Roland Meisel, Michaela Nathrath, Mervi Putkonen, Sarina Ravens, Hans Christian Reinhardt, Elisa Sala, Martin G. Sauer, Clemens Schmitt, Roland Schroers, Nina Kristin Steckel, Ralf Ulrich Trappe, Mareike Verbeek, Daniel Wolff, Rainer Blasczyk, Britta Eiz-Vesper, Britta Maecker-Kolhoff

**Affiliations:** 1 Institute of Transfusion Medicine and Transplant Engineering,; 2 Department of Pediatric Hematology and Oncology,; 3 Cellular Therapy Centre, and; 4 Department of Hematology, Hemostasis, Oncology, and Stem Cell Transplantation, Hannover Medical School, Hannover, Germany.; 5 Department of Pediatrics, University Hospital Schleswig-Holstein, Kiel, Germany.; 6 Pediatric Hematology and Oncology, Klinik für Kinder- und Jugendmedizin, University Children’s Hospital Muenster, Münster, Germany.; 7 University Children’s Hospital Heidelberg, Heidelberg, Germany.; 8 Department of Hematology, Oncology, and Tumor Immunology, Charité–Universitätsmedizin Berlin, Berlin, Germany.; 9 Clinic for Hematology and Oncology, University Medicine Göttingen, Georg August University, Göttingen, Germany.; 10 Pediatric Hematology and Oncology, Department for Pediatrics III, University Hospital Essen, Essen, Germany.; 11 Department of Pediatric Oncology and Hematology, Charité–Universitätsmedizin Berlin, Berlin, Germany.; 12 Department of Hematology, Oncology, Hemostaseology, and Stem Cell Transplantation, University Medical Center RWTH Aachen, and Center for Integrated Oncology Aachen Bonn Cologne Düsseldorf (CIO ABCD), Aachen, Germany.; 13 Department of Pediatrics I, Martin Luther University Halle-Wittenberg, Halle, Germany.; 14 Third Department of Medicine–Haematology, Internal Oncology, and Pneumology, Johannes Gutenberg University Medical Center, Mainz, Germany.; 15 Department for Stem Cell Transplantation, University Medical Center Hamburg, Hamburg, Germany.; 16 Charité-Universitätsmedizin Berlin, Hematology, Oncology, and Tumor Immunology, corporate member of Freie Universität Berlin and Humboldt-Universität zu Berlin, Berlin, Germany.; 17 Group Biology of Malignant Lymphomas, Max Delbrück Center for Molecular Medicine in the Helmholtz Association (MDC), Berlin, Germany.; 18 Experimental and Clinical Research Center (ECRC), a cooperation between the MDC and the Charité, Berlin, Germany.; 19 Division of Pediatric Stem Cell Therapy, Department of Pediatric Oncology, Hematology, and Clinical Immunology, Medical Faculty, Heinrich Heine University Düsseldorf, Düsseldorf, Germany.; 20 Department of Pediatric Oncology, Klinikum Kassel, Kassel, Germany, and Children’s Cancer Research Center and Department of Pediatrics, Klinikum rechts der Isar, Technische Universität München, Munich, Germany.; 21 Department of Hematology and Stem Cell Transplantation, Turku University Hospital, Turku, Finland.; 22 Institute of Immunology, Hannover Medical School, Hannover, Germany.; 23 Department of Hematology and Stem Cell Transplantation, West-German Cancer Center, University Hospital Essen, Essen, Germany.; 24 Department of Internal Medicine III, University Hospital Ulm, Ulm, Germany.; 25 Department of Hematology and Oncology, Knappschaftskrankenhaus University Hospital, Bochum, Germany.; 26 German Posttransplant Lymphoproliferative Disease (PTLD) Study Group, Department of Internal Medicine II–Hematology and Oncology, Evangelisches Diakonie-Krankenhaus, Bremen, Germany.; 27 Clinic and Policlinic for Internal Medicine III, School of Medicine, Technische Universität München, Klinikum rechts der Isar, Munich, Germany.; 28 Department of Internal Medicine III, University Hospital Regensburg, Regensburg, Germany.; 29 German Center for Infection Research (DZIF), Braunschweig, Germany.

**Keywords:** Therapeutics, Adaptive immunity, Immunotherapy, T cells

## Abstract

**BACKGROUND:**

Adoptive transfer of EBV-specific T cells can restore specific immunity in immunocompromised patients with EBV-associated complications.

**METHODS:**

We provide results of a personalized T cell manufacturing program evaluating donor, patient, T cell product, and outcome data. Patient-tailored clinical-grade EBV-specific cytotoxic T lymphocyte (EBV-CTL) products from stem cell donors (SCDs), related third-party donors (TPDs), or unrelated TPDs from the allogeneic T cell donor registry (alloCELL) at Hannover Medical School were manufactured by immunomagnetic selection using a CliniMACS Plus or Prodigy device and the EBV PepTivators EBNA-1 and Select. Consecutive manufacturing processes were evaluated, and patient outcome and side effects were retrieved by retrospective chart analysis.

**RESULTS:**

Forty clinical-grade EBV-CTL products from SCDs, related TPDs, or unrelated TPDs were generated for 37 patients with refractory EBV infections or EBV-associated malignancies with and without a history of transplantation, within 5 days (median) after donor identification. Thirty-four patients received 1–14 EBV-CTL products (fresh and cryopreserved). EBV-CTL transfer led to a complete response in 20 of 29 patients who were evaluated for clinical response. No infusion-related toxicity was reported. EBV-specific T cells in patients’ blood were detectable in 16 of 18 monitored patients (89%) after transfer, and their presence correlated with clinical response.

**CONCLUSION:**

Personalized clinical-grade manufacture of EBV-CTL products via immunomagnetic selection from SCDs, related TPDs, or unrelated TPDs in a timely manner is feasible. Overall, EBV-CTLs were clinically effective and well tolerated. Our data suggest EBV-CTL transfer as a promising therapeutic approach for immunocompromised patients with refractory EBV-associated diseases beyond HSCT, as well as patients with preexisting organ dysfunction.

**TRIAL REGISTRATION:**

Not applicable.

**FUNDING:**

This study was funded in part by the German Research Foundation (DFG, 158989968/SFB 900), the Deutsche Kinderkrebsstiftung (DKS 2013.09), Wilhelm-Sander-Stiftung (reference 2015.097.1), Ellen-Schmidt-Program of Hannover Medical School, and German Federal Ministry of Education and Research (reference 01EO0802).

## Graphical abstract

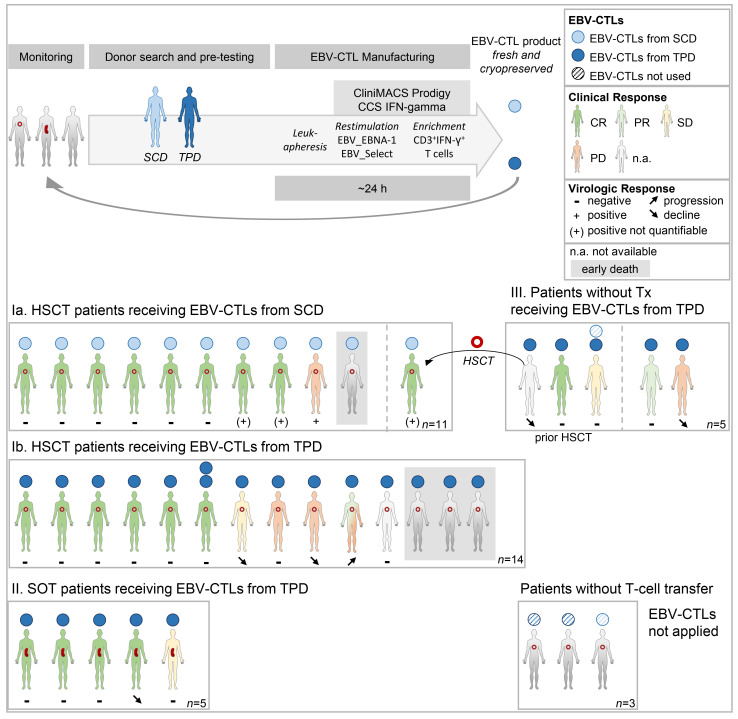

## Introduction

Morbidity and mortality in patients with hematopoietic stem cell transplant (HSCT) and solid organ transplant (SOT) are frequently caused by graft rejection or graft-versus-host disease (GvHD) and increased by infectious complications due to delayed immune reconstitution or immunosuppressive medication (1). EBV is a γ-herpesvirus that infects more than 90% of the population worldwide during childhood and persists throughout life within the B cell compartment. Strong CD4^+^ and CD8^+^ T cell responses directed against various lytic and latent EBV proteins control EBV reactivation and usually prevent EBV-associated diseases in healthy individuals (2). However, in immunocompromised patients, infection and reactivation as well as the development of high-grade secondary malignancies are severe complications.

The most common EBV-associated posttransplant malignancy is posttransplant lymphoproliferative disease (PTLD), representing both clinically and histopathologically heterogeneous lymphoproliferations (3–7). The overall incidence of PTLD after allogeneic HSCT is less than 2% but was shown to increase after transplantation with T cell–depleted and/or HLA-mismatched grafts (e.g., ≤24%) (8, 9). During the phase of immune reconstitution, e.g., the first 6–12 months after HSCT, incidences can reach up to 40% (10–12).

In SOT, EBV-seronegative transplant recipients with EBV-seropositive donors and those experiencing primary EBV infection under posttransplant immunosuppression consequently carry the highest risk of developing EBV-associated PTLD (3, 13–15). Incidences vary from 1% to 20% depending on the organ type; incidences are high during the first year after transplant, with almost all tumors being EBV associated, but PTLD can occur any time during the period of immunosuppression (3, 5, 16). The type of organ graft — i.e., with respect to its amount of lymphoid tissue as well as the level of immunosuppression needed to maintain immune tolerance — represents another distinctive factor, with highest incidences (≤20%) detected in lung, small bowel, or multiple organ grafts (14, 15, 17–19). Further risk factors for PTLD development are the patient’s age at transplantation (especially <18 and >60 years), the disease initially leading to transplantation, a previous splenectomy, a second transplantation, coinfection with CMV, polymorphisms in cytokine genes, the intensity and duration of the immunosuppressive regimen, the HLA type and extent of HLA mismatch between donor and recipient, whether T cell–depleting antibodies (e.g., anti–thymocyte globulin [ATG], alemtuzumab) are being administered, as well as the coexistence of multiple risk factors (10, 13–15, 17, 20, 21). The incidence of PTLD after HSCT in patients with no risk factors was found to be low (0.2%), but it significantly increased (8.1%) if there were 3 or more risk factors (10).

Treatment of PTLD includes reduction of immunosuppressive medication as tolerated, immunotherapy, and cytotoxic chemotherapy. However, therapy is often complicated by side effects, and severe complications are foreseeable in patients with preexisting organ dysfunction (3, 11, 22).

Besides reduction of immunosuppression, the 2 main immunotherapeutic approaches to treat EBV-associated PTLDs are (i) treatment with mAbs (e.g., rituximab) against the B cell surface molecule CD20 to eliminate EBV-infected B cells; and (ii) adoptive transfer of functional EBV-specific cytotoxic T lymphocytes (EBV-CTLs) from healthy related or unrelated donors. Treatment with anti-CD20 mAb is often associated with a risk of infection in immunosuppressed patients and is sometimes ineffective due to low or absent CD20 expression and antigen loss during treatment.

Adoptive T cell therapy appears to be an attractive therapeutic option. First developed in the 1990s, autologous — or stem cell donor–derived (SCD-derived) — EBV-CTL lines were generated by repetitive in vitro stimulation with antigen-bearing cells (23). More recently, short-term expansion strategies were developed to generate either SCD-derived (24) or partially HLA-matched third-party donor–derived (TPD-derived) EBV-CTLs within 2 weeks (25–29). Some of these studies targeted multiple viral infections (28, 29). In all studies, EBV-CTL infusions were in general well tolerated and effective in the majority of patients.

Although this treatment approach is attractive, it has several limitations. Generation of EBV-CTLs by in vitro culture is time-consuming and relies on specialized cell growth facilities. Feuchtinger and colleagues developed a different strategy: selecting EBV-CTL directly from peripheral blood by magnetic separation based on EBV-specific cytokine secretion (30). For immunomagnetic selection of EBV-CTLs, a donor with sufficient frequencies of EBV-specific T cells is needed, a requirement that often fails in HSCT patients and is impossible to achieve from deceased organ donors. Third, transfer of bystander cells may confer GvHD or allograft rejection.

The current study was designed to retrospectively analyze our program of personalized EBV-CTL manufacturing via immunomagnetic selection from SCDs, related TPDs, or unrelated TPDs from an allogeneic T cell donor registry (alloCELL) established at the Institute of Transfusion Medicine and Transplant Engineering, Hannover Medical School. In total, 40 clinical-grade EBV-CTL products were manufactured for administration in 37 immunocompromised patients with and without transplantation history. Data were analyzed with respect to manufacturing time, cell numbers, and transfer frequency; infusion-related side effects; influence on GvHD; as well as clinical, immunological, and virologic outcome.

## Results

### Patient cohort

**Figure 1:**
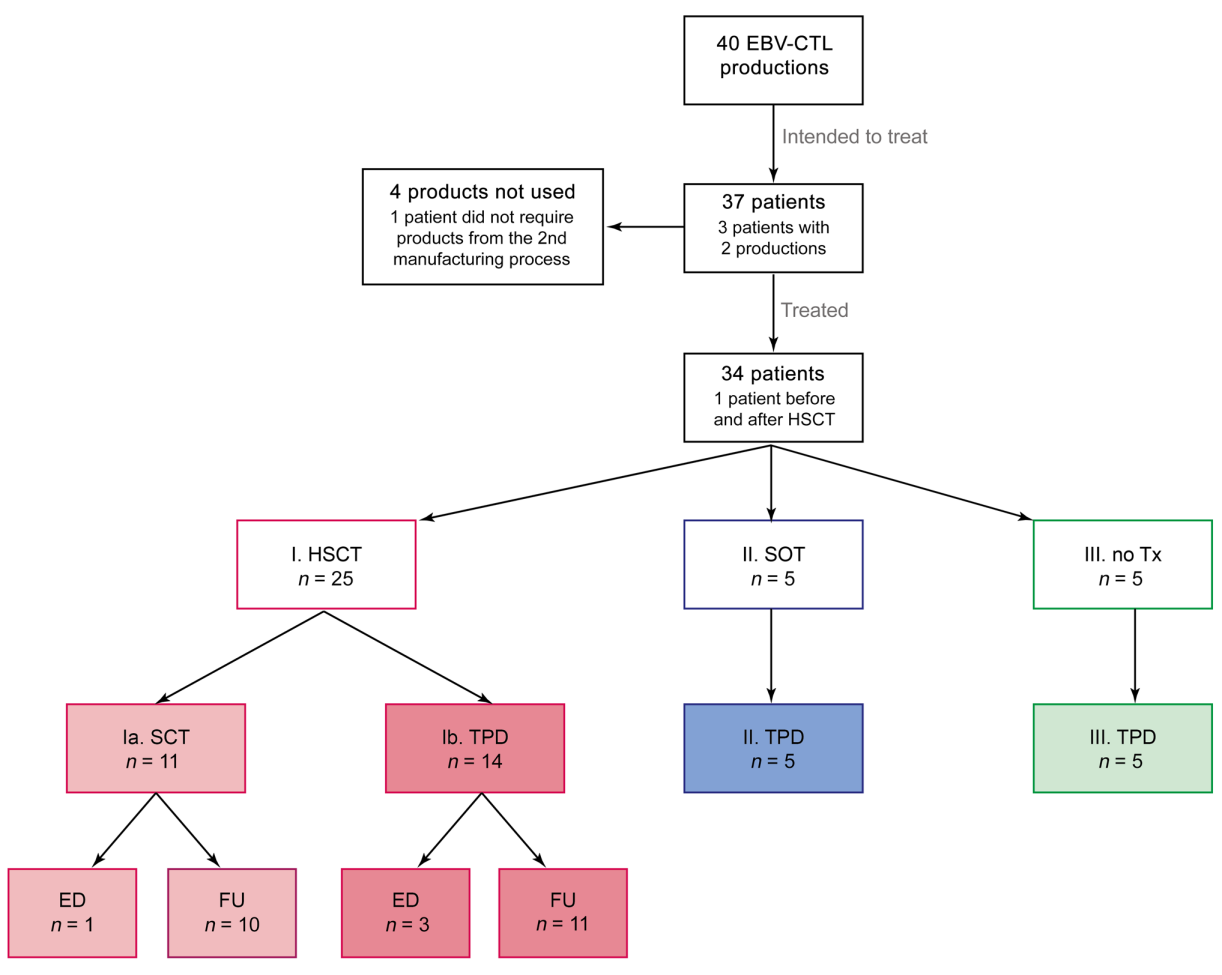
Patient cohort for planned EBV-CTL transfer. EBV-CTL productions and intended/treated patient population. One patient received EBV-CTLs before and after HSCT and is therefore recorded in groups IB and IIIA. One patient in group Ia received EBV-CTLs from 2 separate productions from the same donor. pt, patient; Tx, transplantation; ED, early death; FU, follow up.

A total of 40 EBV-CTL products were manufactured between May 2015 and July 2019 for 37 patients with refractory EBV infections or EBV-associated malignancies from 21 different hospitals (20 in Germany, 1 in Finland) intended to receive EBV-CTLs (Figure 1 and Supplemental Table 1, A and B; supplemental material available online with this article; https://doi.org/10.1172/JCI163548DS1). Three patients (nos. 1/3, 24/36, and 28) have been reported before but were added to this series for completeness (31–33). The median age of patients was 37 years (range 2–73 years); 26 patients were male and 11 female (Table 1). Five patients had no transplant history before the planned EBV-CTL transfer; 4 of these had EBV-associated malignancies, and 1 had chronic EBV infection without lymphoproliferation due to an inborn immunodeficiency syndrome. Three of these patients intended to undergo allogeneic HSCT after EBV-CTL transfer, and in 2 of them a second transfer from a different manufacturing process after HSCT was planned (no. 24/36, second transfer received; no. 33/40, second transfer not received). Twenty-eight patients were scheduled to receive EBV-CTLs after HSCT, including the 2 patients mentioned above. Indications for HSCT were malignancy (*n* = 18) and nonmalignant disease (*n* = 10) (Supplemental Table 1A). One of the HSCT patients received EBV-CTLs from 2 different manufacturing processes (Supplemental Table 1, A and B, no. 1/3; both from the same unrelated TPDs [alloCELL]). For 2 patients (nos. 24/36, 33/40), EBV-CTL preparations from 2 different donors were manufactured, while 34 patients were scheduled for a single T cell preparation.

Five patients had a history of SOT prior to EBV-CTL transfer (1 heart, 2 kidney, and 2 liver transplants). All SOT patients received EBV-CTLs from TPDs for EBV-associated PTLD; none of them experienced malignant disease before SOT.

### Donor selection based on serostatus, HLA match, and EBV-specific T cell frequency

#### Donor search and donor pretesting.

Selection of EBV-seropositive donors suitable for generation of EBV-CTLs was based on HLA match, EBV serostatus, and the frequency of EBV-specific T cells as determined by IFN-γ cytokine secretion assay (CSA), which is an analog to the clinical-scale CliniMACS Cytokine Capture System (CCS) IFN-gamma process. Patients prior to or after HSCT can be treated with EBV-CTLs isolated from SCDs if these are present in the donor at a sufficient frequency. Alternatively, (partially) HLA-matched related or unrelated TPDs can potentially serve as EBV-CTL donors, which is routinely done in the case of patients with SOT or no transplantation history. We here report data from manufacture of 40 EBV-CTL products (Supplemental Table 1, A and B). In 13 patients, the SCDs served as donors of EBV-CTLs (matched sibling donors [MSDs], *n* = 2; matched unrelated donors [MUDs], *n* = 9; haploidentical, *n* = 2); for all other patients (67.5%), EBV-CTLs were manufactured from TPDs (related, *n* = 9; unrelated [alloCELL], *n* = 18).

**Figure 2:**
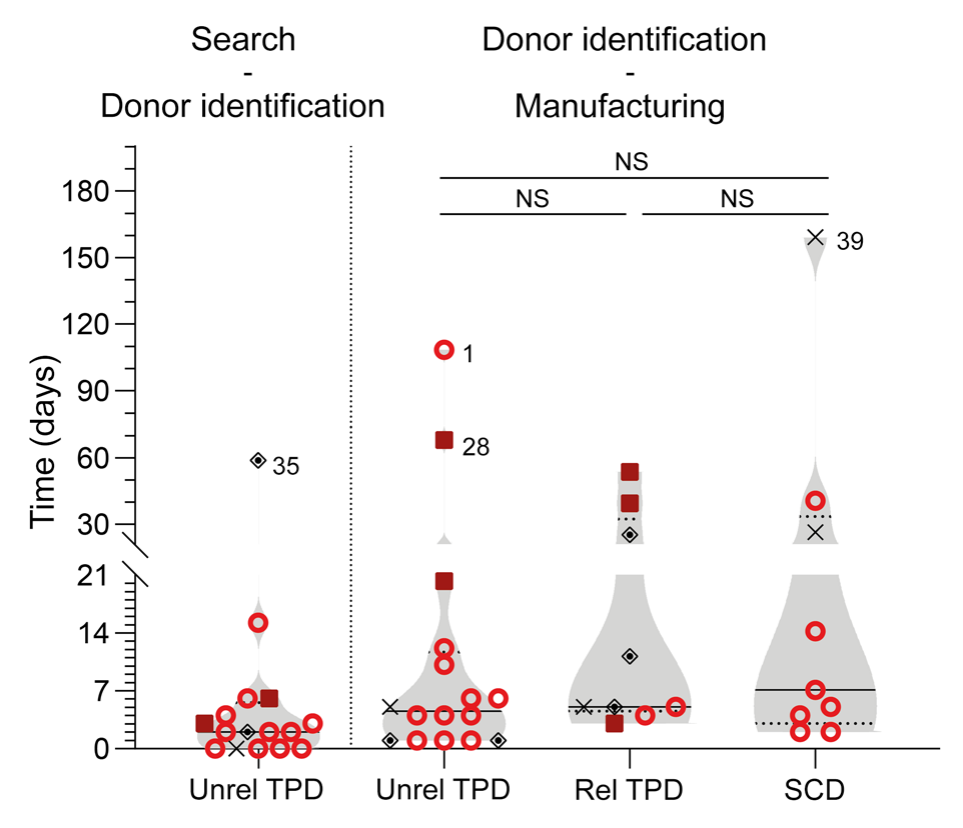
Time between donor search, identification, and manufacturing. Left: Time between donor search and identification for unrelated (unrel) TPDs (left); right: time between donor identification and start of manufacture for each donor origin. Statistical significance was calculated using Kruskal-Wallis test, followed by Dunn’s multiple-comparisons test. NS, not significant, *P* > 0.05. rel, related. Violin plots show median; each symbol represents 1 patient. Red circles, HSCT patients with HSCT (groups Ia/Ib); red squares, patients with SOT (group II); black diamonds, no Tx (group III); black X’s, EBV-CTLs not applied.

The alloCELL T cell donor registry records high-resolution HLA types and virus-specific T cell (VST) frequencies from more than 3,500 healthy volunteers; 18 of 40 manufacturing processes from the current series were performed with cells from donors identified in this noncommercial registry. For 1 patient (no. 1/3), 2 EBV-CTL products were manufactured from the same donor. Thus, in total, 17 donor searches were performed, and the results were provided to the requesting clinic within 24–48 hours. The median number of HLA-matched, EBV-seropositive potential donors identified for each patient was 3 (range 1–8 potential T cell donors; data not shown). All unrelated TPDs were high-resolution typed in HLA-A, -B, -C, -DR, and -DQ. However, for an individual to be suited as an EBV-CTL donor, we required at least a 3/6 HLA match in HLA-A, -B, and -DR, with at least one match in class I and class II alleles each, which applied to all donor-recipient pairs (Table 2). On high-resolution HLA typing, matching was at least 4 of 10 (in 1 patient) and up to 8 of 10 (in 1 patient), with the majority of matches being between 5 of 10 and 7 of 10 (details in Table 2). The median time between donor search result for unrelated TPD and donor identification was 2 days (Figure 2, *n* = 16). The median time between donor pretesting result and start of the manufacturing process was 5 days and did not significantly differ between unrelated TPDs (5 days), related TPDs (5 days), and SCDs (7 days) (Figure 2, *n* = 34). T cell manufacturing is an overnight process. Prolonged times between donor identification and manufacturing resulted from individual pretreatment regimens with chemo/immunotherapy (patients 1, 28, 35, 39); these cases are identified by number in Figure 2, with additional information in Supplemental Table 1, A and B. The HLA match between patients and related TPDs is shown in Table 2.

**Figure 3:**
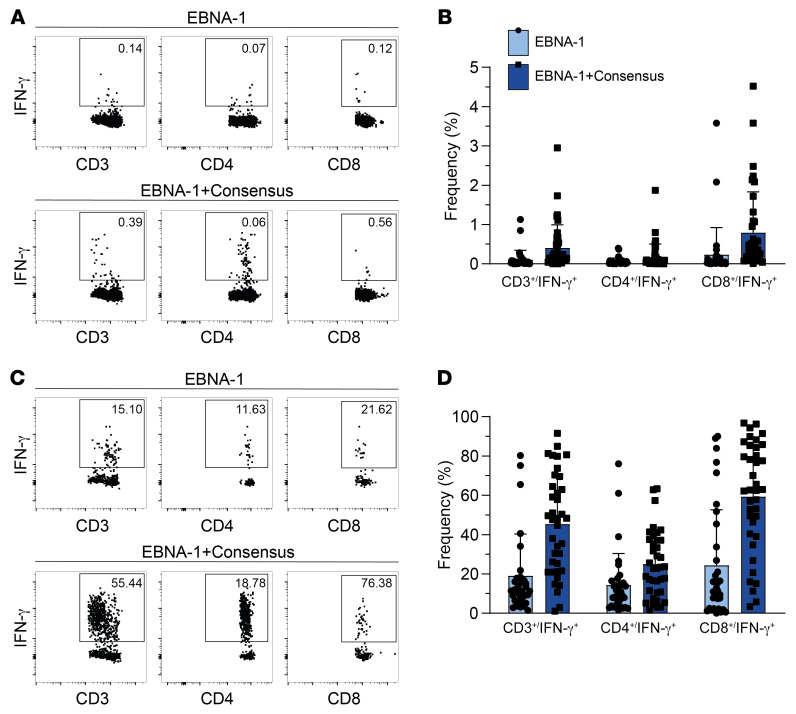
Frequencies of EBV-specific T cells in T cell donors before and after magnetic enrichment by IFN-γ CSA. Stimulation of donor PBMCs was done with PepTivator EBNA-1 alone (*n* = 34) and a combination of PepTivator EBNA-1 and PepTivator Consensus (*n* = 38). Differences in the numbers of donors tested are due to the amount of PBMCs obtained, which did not allow for testing the frequency of EBNA‑1–specific T cells alone in 4 of 38 donors. Exemplary FACS plots pregated on viable CD3^+^, CD3^+^CD4^+^ (middle), and CD3^+^CD8^+^ (right) lymphocytes. (**A** and **B**) Representative FACS plots and summarizing graphs show frequencies of IFN‑γ^+^ cells among CD3^+^, CD4^+^, and CD8^+^ T cells before magnetic enrichment as indicated. (**C** and **D**) Representative FACS plots and summarizing graphs show IFN‑γ^+^ cells among CD3^+^, CD4^+^, and CD8^+^ T cells after magnetic enrichment as indicated. Bar graphs show mean + SD, and each dot represents data from one donor.

For SCDs as well as related TPDs, EBV serostatus was determined prior to donor pretesting. Only EBV-seropositive donors were analyzed with respect to frequencies of EBV-specific T cells (34). In order to determine the starting frequencies of therapeutically relevant EBV-specific T cells (30), we performed pretesting by stimulation using an EBV PepTivator EBNA-1 alone and in combination with a PepTivator Consensus (analogous to GMP PepTivator EBV Select used in clinical-grade manufacturing). Unstimulated cells served as negative control (NC), and values obtained from the NC were subtracted from preenrichment values. For all donors tested (*n* = 38), stimulation with EBNA-1 and Consensus in combination was performed, while for 4 donors, the amount of PBMCs obtained did not allow for determination of EBNA-1–specific T cell frequencies alone. The mean frequency of IFN-γ^+^ T cells upon stimulation with EBNA-1 was 0.11% (CD3^+^), 0.05% (CD4^+^), and 0.24% (CD8^+^); and the mean frequency of CD3^+^IFN-γ^+^, CD4^+^IFN-γ^+^, and CD8^+^IFN-γ^+^ T cells increased to 0.41%, 0.17%, and 0.80%, respectively, upon stimulation with the combination of EBNA-1 and Consensus (Table 3 and Figure 3, A and B). Following magnetic enrichment, the mean frequency of IFN-γ^+^ T cells upon stimulation with EBNA-1 was 19.13% (CD3^+^), 14.47% (CD4^+^), and 24.41% (CD8^+^), which increased to 45.36%, 25.05%, and 59.39%, respectively, upon stimulation with the combination of EBNA-1 and Consensus (Table 3 and Figure 3, C and D).

### Manufacturing of clinical-grade EBV-CTL products

**Figure 4:**
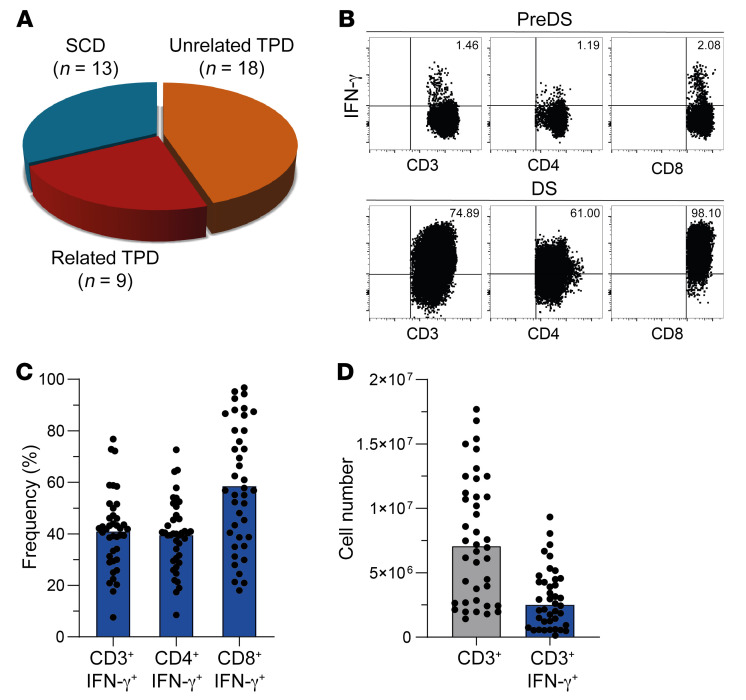
Clinical-grade EBV-CTL manufacturing. Enrichment of IFN‑γ–secreting, EBV-specific CD3^+^, CD4^+^, and CD8^+^ T cells after incubation with GMP-grade PepTivators EBV EBNA-1 and EBV Select in combination using the CliniMACS CCS and CliniMACS Plus or Prodigy device. (**A**) Donor origin. (**B**) Representative FACS plots. Gates were set according to fluorescence minus one (FMO) control. preDS, drug substance before magnetic enrichment; DS, drug substance after magnetic enrichment. (**C** and **D**) Frequencies and numbers of total CD3^+^ and IFN-γ–secreting, EBV-specific CD3^+^, CD4^+^, and CD8^+^ T cells after stimulation with GMP-grade PepTivators EBNA-1 and EBV Select and enrichment using the CliniMACS CCS and CliniMACS Plus or Prodigy device. Bar graphs depict mean (**C**) or median (**D**), and each dot represents data from 1 manufacturing process (*n* = 40).

In total, 40 clinical-grade EBV-CTL products were generated for 37 patients. Thirteen EBV-CTLs were derived from the respective SCDs, 18 from unrelated TPDs, and 9 from related TPDs (Figure 4A). Manufacturing was performed using MACS GMP PepTivator EBV_EBNA-1 in combination with MACS GMP PepTivator EBV_Select and the CliniMACS CCS together with the CliniMACS Plus (*n* = 13) or CliniMACS Prodigy (*n* = 27) device (Supplemental Table 2) as described previously (35). In brief, 1 × 10^9^ donor white blood cells obtained via leukapheresis were restimulated with MACS GMP PepTivator EBV_EBNA-1 and MACS-GMP PepTivator EBV_Select for 4 hours, followed by immunomagnetic selection of IFN-γ–secreting cells. The total T cell numbers (CD3^+^ and CD3^+^IFN‑γ^+^) obtained were significantly higher when CliniMACS Prodigy was used compared with CliniMACS Plus (CD3^+^ *P* < 0.0001, CD3^+^IFN‑γ^+^ *P* = 0.0014; Supplemental Table 2 and data not shown). For all processes (*n* = 40), the mean viability of the generated EBV-CTLs was 70.5% and the mean frequency of CD3^+^IFN‑γ^+^ T cells was 40.9%, which constituted 39.5% and 58.8% IFN‑γ^+^ cells among CD4^+^ and CD8^+^ subsets, respectively (Table 4 and Figure 4, B and C). The median number of total CD3^+^ cells was 7.07 × 10^6^, corresponding to a median number of 2.52 × 10^6^ CD3^+^/IFN‑γ^+^ T cells (Table 4 and Figure 4D). There was no significant difference between SCDs, related TPDs, and unrelated TPDs with respect to T cell numbers and purity in the final EBV-CTL products (Table 4).

In contrast to the overlapping peptide pool PepTivator EBNA-1, PepTivator EBV Consensus contains 32 MHC class I–restricted and 11 MHC class II–restricted peptides, which are derived from 15 lytic and latent EBV proteins. The HLA-A and HLA-B alleles involved in recognition of these peptides as well as their representation in 31 of the EBV-CTL donors are listed in Table 5. Homozygous alleles were considered only once. The HLA restrictions and epitope specificities of the administered T cells were not defined. Manufacturing was performed using a combination of PepTivator EBV EBNA1 and PepTivator EBV Select. Hence, it can be assumed that the HLA coverage of the obtained CD3^+^IFN-γ^+^ T cells was not restricted to those HLA alleles covered by PepTivator EBV Select.

### Patient follow-up

Three patients did not receive the EBV-CTL product because of death (*n* = 2; nos. 37, 38) or cure (*n* = 1; no. 39) before the end of the manufacturing process. A fourth patient (no. 33/40) received TPD-derived EBV-CTLs (no. 33) before HSCT but no longer required the already-produced SCD-derived EBV-CTLs (no. 40) after HSCT. These 4 products were excluded from the analysis of clinical effects and side effects. For all patients included in the analysis, the median CD3^+^ T cell number for the first EBV-CTL transfer was 2.5 × 10^4^/kg (range 5 × 10^3^ to 2.2 × 10^5^/kg) and the median CD3^+^ T cell number for all transfers was 4.2 × 10^4^/kg (range 5 × 10^3^ to 2.2 × 10^5^/kg). The mean purity of transferred EBV-CTL products measured by percentage of CD3^+^IFN-γ^+^ cells was 41.8% (range 17.7%–76.8%), corresponding to a median number of 7.9 × 10^3^/kg CD3^+^IFN-γ^+^ T cells (range 2.2 × 10^3^ to 9.8 × 10^4^/kg) for the first EBV-CTL transfer. Median follow-up was 34.5 months for all patients and 49.5 months (range 11–77) for the patients who were alive at last follow-up. Details are shown in Supplemental Table 1, A and B.

#### HSCT patients with EBV-CTLs from SCDs (group Ia).

For 11 HSCT patients who received the EBV-CTL product after HSCT, the SCD served as EBV-CTL donor. One patient died within 3 weeks after EBV-CTL transfer (multiorgan failure) and was excluded from the long-term follow-up evaluation (“early death,” no. 18). All patients with B cell PTLD had received prior treatment with rituximab (*n* = 9) or chemotherapy (*n* = 2); 1 patient with NK/T cell non-Hodgkin lymphoma (NKT-NHL) received PD1-blockade in parallel to EBV-CTL treatment (no. 23). The median number of EBV-CTL transfers in these patients was 1 (range 1–5); the median CD3^+^ T cell number for the first EBV-CTL transfer was 2.5 × 10^4^/kg (range 1 × 10^4^ to 2.2 × 10^5^/kg); the mean percentage of CD3^+^IFN-γ^+^ T cells was 33.8%, corresponding to a median number of 8.5 × 10^3^/kg CD3^+^IFN-γ^+^ T cells. One of these patients received EBV-CTLs before transplantation for EBV-associated encephalitis and received a total of 14 EBV-CTL administrations (9 before HSCT from an unrelated TPD, no. 36; 5 in parallel to or after HSCT from the SCD, no. 24). Nine of 10 patients achieved complete response (CR) following EBV-CTL transfer (Table 6). In 6 of these, EBV in peripheral blood became undetectable by PCR (nos. 16, 19, 20, 21, 22, 26; 16, and 21 already had negative EBV-PCR before transfer of EBV-CTLs), whereas in 3 patients EBV-PCR remained positive (nos. 17, 23, 24). Details for EBV-PCR load monitoring in patients with serial measurements are shown in Supplemental Figure 1. Of the patients with CR, 3 died due to infections other than EBV (nos. 19, 22, 23). One patient had progressive disease (PD) irrespective of EBV-CTL transfer and died 4 weeks after transfer of EBV-CTL due to progression of EBV-related PTLD (no. 25).

No graft failure was detected after EBV-CTL transfer. Three patients in this group of 10 had GvHD before administration of EBV-CTLs, 2 of them were free of GvHD after EBV-CTL transfer (nos. 16, 19), and the third developed chronic GvHD without new GvHD symptoms (no. 22). Of the 7 patients without preexisting GvHD, 3 developed GvHD after EBV-CTL transfer. Two of these had mild to moderate GvHD, which in one of them began directly after reduction of immunosuppression (nos. 17, 20); the third patient developed grade III GvHD of the liver, skin, and oral mucosa (no. 23). This patient received not only EBV-CTLs but also donor lymphocyte infusions (DLIs) and pembrolizumab in parallel. The treating physician did not attribute GvHD to EBV-CTL transfer. All 3 patients with new GvHD after EBV-CTL transfer had received only a single transfer; transferred cell numbers were 3.04 × 10^4^/kg, 2.5 × 10^4^/kg, and 5.0 × 10^4^/kg CD3^+^ cells (corresponding to 5.4 × 10^3^/kg, 1.1 × 10^4^/kg, and 2.0 × 10^4^/kg CD3^+^IFN-γ^+^ T cells), respectively.

#### HSCT patients with EBV-CTLs from TPDs (group Ib).

Fourteen HSCT patients received EBV-CTLs from TPDs after HSCT; one of them received EBV-CTLs from 2 different manufacturing processes with the same unrelated TPD (no. 1/3). Two patients received EBV-CTLs from related TPDs (nos. 14, 15); unrelated TPDs were used for the other 12 patients. Three patients died within 3 weeks after EBV-CTL transfer (progression of EBV-associated encephalitis, no. 2; progression of AML and PTLD, no. 10; multiorgan failure, presumably EBV-related, no. 5). These patients were excluded from the long-term follow-up evaluation (assigned to the “early death” group). All patients had received rituximab prior to EBV-CTL transfer; in 3 patients, additional chemotherapy had been administered. Details are shown in Table 6 and Supplemental Table 1, A and B.

The median number of EBV-CTL transfers in the remaining 11 patients was 2 (range 1–6), the median CD3^+^ T cell number for the first EBV-CTL transfer was 1.75 × 10^4^/kg (range 5 × 10^3^ to 3.69 × 10^4^/kg); and the mean percentage of CD3^+^IFN-γ^+^ T cells was 45.9%, corresponding to a median number of 5.0 × 10^3^/kg CD3^+^IFN-γ^+^ T cells. Six of 10 patients with outcome data available had a CR after EBV-CTL transfer, with resolution of all symptoms; in these cases, EBV became undetectable by PCR in peripheral blood (no. 1/3, 6, 7, 11, 12, 14; in one patient, no. 7, EBV-PCR remained low-positive in cerebrospinal fluid). Details for EBV-PCR load monitoring in patients with serial measurements are shown in Supplemental Figure 1. Four of the patients with CR survived (nos. 1/3, 11, 12, 14); the remaining 2 patients (nos. 6, 7) died from causes unrelated to EBV. In 1 patient (no. 13), the EBV-associated symptoms remained stable after EBV-CTL transfer; the EBV-PCR remained positive but revealed a decrease in viral load. This patient died from causes unrelated to EBV. Three patients had PD following EBV-CTL transfer (nos. 8, 9, 15), although 1 of them (no. 9) had a negative EBV-PCR; all of them died (2 of them EBV-related causes; the third because of multiorgan failure otherwise not classified). For the fourth patient (no. 4), no data were available concerning the clinical response to EBV-CTLs; although the EBV-PCR was negative, this patient died for reasons unrelated to EBV.

No graft failure occurred after EBV-CTL transfer. Nine of 11 patients had a history of GvHD before EBV-CTL transfer. In 2 of these (nos. 7, 14), GvHD persisted at the same level after EBV-CTL transfer; and in one case, acute GvHD developed into chronic GvHD (no. 4; this patient died due to chronic GvHD). Two of the patients with preexisting GvHD developed new GvHD symptoms following EBV-CTL transfer, but one of them received DLI and the other nivolumab due to recurrence of underlying malignancy concurrently with transfer of EBV-CTLs (nos. 6, 15). Preexisting GvHD resolved in 4 cases after transfer of EBV-CTLs (nos. 1/3, 9, 12, 13). Neither of the 2 patients who were free of GvHD before EBV-CTL transfer developed de novo GvHD thereafter (nos. 8, 11).

#### SOT patients (group II).

Five patients had a history of SOT and received EBV-CTLs for refractory or high-risk EBV-related PTLD. Four patients with CD20^+^ PTLD had received rituximab; all patients had received chemotherapy prior to EBV-CTL transfer, which resulted in CR before EBV-CTL transfer in 2 of 5 patients. Related (*n* = 3, nos. 27, 29, 30; Supplemental Table 1, A and B) or unrelated TPDs (*n* = 2, nos. 28, 31; Supplemental Table 1, A and B) served used as EBV-CTL donors. The median number of EBV-CTL transfers was 3 (range 1–5); the median CD3^+^ T cell number for the first EBV-CTL transfer was 2.5 × 10^4^/kg (range 1 × 10^4^ to 4.2 × 10^4^/kg); the mean percentage of CD3^+^IFN-γ^+^ T cells was 35.5%, corresponding to a median number of 9.4 × 10^3^/kg CD3^+^IFN-γ^+^ T cells. Four patients showed CR, and 3 of them had negative EBV-PCR following EBV-CTL transfer (nos. 28, 29, 30); however, these 3 patients already had negative EBV-PCR before transfer of EBV-CTLs, and the fourth patient with CR still had a positive EBV-PCR (no. 31). The remaining (fifth) patient showed stable disease (SD) clinically, but the EBV-PCR turned negative and PET/CT showed a complete metabolic response (no. 27). None of the SOT patients developed GvHD or experienced graft loss after EBV-CTL transfer, and all of them were alive at last follow-up.

#### Patients without a history of transplantation (group III).

Five patients received EBV-CTLs without any, or prior to, transplantation for either refractory EBV infection in suspected/verified immunodeficiency (*n* = 3; nos. 33, 34, 36) or EBV-related lymphomatoid malignancy (*n* = 2; nos. 32, 35; Supplemental Table 1, A and B). Partially HLA-matched related (*n* = 3; nos. 32, 33, 34) or unrelated TPDs (*n* = 2; nos. 35, 36) served as EBV-CTL donors. In these patients, the median number of EBV-CTL transfers was 3 (range 2–9); the median CD3^+^ T cell number for first EBV-CTL transfer was 1.0 × 10^4^/kg (range 1.0 × 10^4^ to 2.5 × 10^4^/kg); and median CD3^+^ T cell number for all EBV-CTL transfers was 4.3 × 10^4^/kg. The mean percentage of CD3^+^IFN-γ^+^ T cells was 51.1%, corresponding to a median number of 5.9 × 10^3^/kg CD3^+^IFN-γ^+^ T cells for the first EBV-CTL transfer. The 3 patients with immunodeficiency underwent HSCT afterward; of these, 1 patient received EBV-CTLs after transplantation as well (no. 36; see “HSCT patients with EBV-CTLs from TPDs (group Ib)”; among the patients without a history of transplantation (group III), only the transfers performed before HSCT were considered.

After treatment, 1 of the 5 patients (no. 32) showed PD, and their EBV-PCR remained positive; this patient died due to progression of EBV-associated lymphoproliferation. A second patient (no. 33) had SD with increasing EBV load despite EBV-CTL transfer; however, the EBV-PCR became negative after HSCT, and the patient no longer required EBV-related treatment after transplant. A third patient (no. 34) initially showed a partial clinical response (PR) and a decrease in EBV load, and achieved CR as well as a negative EBV-PCR after HSCT. A fourth patient (no. 35) had a negative EBV-PCR before transfer of EBV-CTLs; this patient showed a PR clinically, whereas by PET/CT, a complete metabolic response was seen. A fifth patient (no. 36) had CR, but the EBV-PCR remained positive; therefore, this patient received EBV-CTLs after HSCT as well. Except for patient no. 32, all patients were alive at last follow-up. None of the patients developed GvHD following EBV-CTL transfer.

### Detection of EBV-specific T cells in patients’ blood

**Figure 5:**
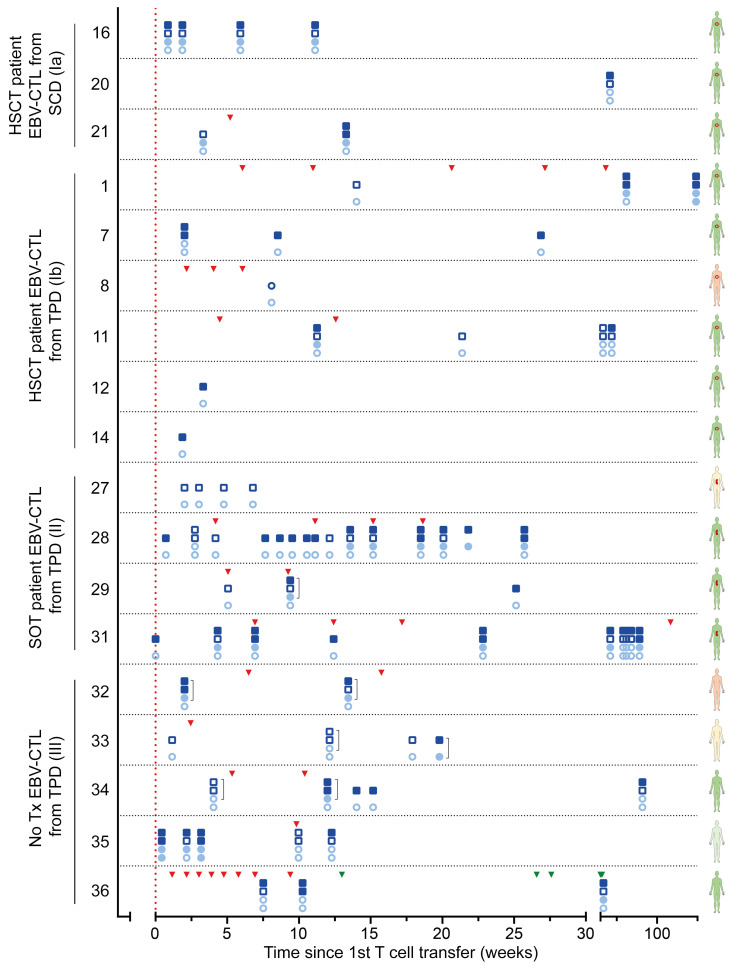
Immune monitoring in individual patients by IFN-γ ELISpot assay. Peripheral blood samples obtained from patients (identification number indicated on the *y* axis) at different time points after first EBV-CTL transfer (indicated on the *x* axis; in weeks) were subjected to IFN-γ ELISpot assay using EBV_EBNA-1 (light blue) and EBV_Consensus (dark blue) to restimulate EBV-specific memory T cells. Lower circles indicate results from direct EBV_EBNA-1 ELISpot; upper circles indicate results from EBV_EBNA-1 ELISpot after expansion. For each patient, lower circles indicate results from direct EBV_EBNA-1 ELISpot; upper circles indicate results from EBV_EBNA-1 ELISpot after expansion. For each patient, lower squares indicate results from direct EBV_Consensus ELISpot, upper squares indicate results from EBV_Consensus ELISpot after expansion. Square brackets (]) indicate combined stimulation with both EBV_EBNA-1 and EBV_Consensus. Empty symbols indicate that no specific T cells were detected, while filled symbols indicate that specific T cells were detected. The vertical dashed line and triangles indicate time points of T cell transfer. No. 36: green triangles indicate T cell transfer from a second manufacturing process (no. 24). Schematics of human figures on the right indicate the type of transplant (red circle: HSCT; kidney shape: SOT) and clinical response (green: CR; light green: PR; yellow: SD; orange: PD).

**Figure 6:**
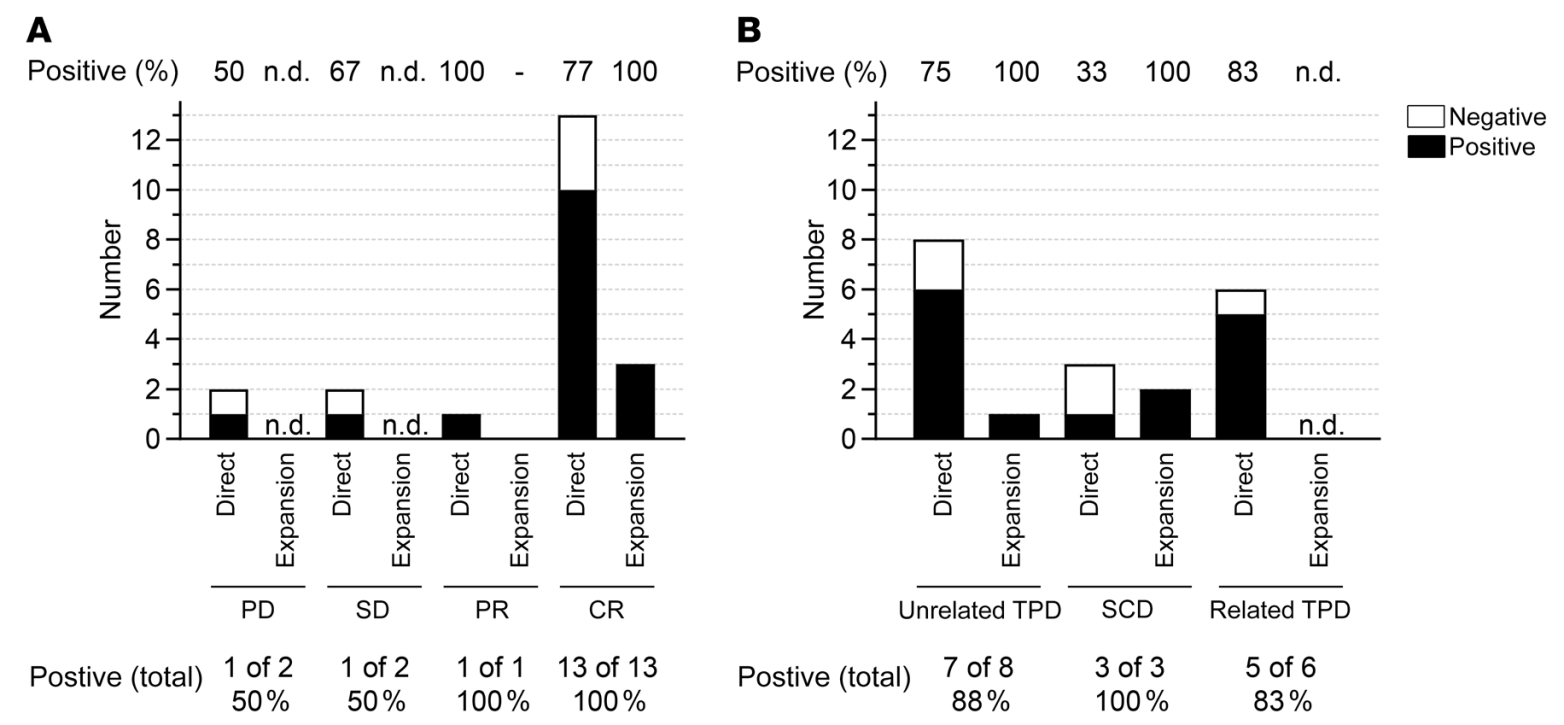
T cell monitoring results. Detection of IFN‑γ–secreting T cells in patient PBMCs after stimulation with PepTivator EBV_EBNA-1 or EBV Consensus using IFN-γ ELISpot assay. Positive: Spots in at least one of the EBV peptide pools. Negative: No spots. Results shown for “Expansion” include only those patients without detectable EBV-CTLs via direct IFN-γ ELISpot assay. Numbers and frequencies (bottom) indicate in how many patients among the total tested EBV-CTLs were detected via either direct IFN-γ ELISpot or after expansion. (**A**) T cell monitoring results based on clinical response. (**B**) T cell monitoring results based on donor origin. n.d. not determined.

EBV-specific T cell monitoring in PBMCs after EBV-CTL transfer was performed for 18 of 37 patients. Monitoring results and time points of T cell transfers for individual patients are shown in Figure 5 and summarized in Figure 6. EBV-specific T cell responses were detected in 13 of these (72%) directly ex vivo by using an IFN-γ enzyme-linked immune absorbent spot (ELISpot) assay (Figure 6). PBMCs from 3 of the 5 patients in whom EBV-specific T cells were undetectable via direct ELISpot assay were restimulated once using EBV peptide pools and expanded for 7 days in the presence of low-dose IL-2. This allowed for more sensitive detection of low-frequency VSTs and at the same time indicated their functionality as defined by the ability to proliferate and secrete IFN-γ upon antigen recognition. In all of them, EBV-specific T cells were detected after expansion, resulting in a total EBV-specific T cell detection rate of 89% (3 of 4 positive after expansion, 16 of 18 positive in total). In these 18 patients, the median time between first EBV-CTL application and monitoring for EBV-specific T cells in recipient blood was 3 weeks.

Among the 20 patients with clinical CR, monitoring was performed for 13 (Figure 6A). Of these, 10 patients had detectable EBV-specific T cells (77%, 3 of 3 positive after expansion, total 100%). The patient showing PR was monitored and had detectable EBV-specific T cells (100%). Of the 3 patients showing SD, 2 were monitored, and in one of them, EBV-specific T cells were detected (50%, no expansion performed). Two of the 4 patients with PD were monitored, and one of them had detectable EBV-specific T cells (50%, no expansion performed). In summary, we were able to detect EBV-specific T cell responses in all patients with PR or CR after EBV-CTL transfer.

For analysis of EBV-specific T cell responses in patients based on donor origin, one patient was excluded because they received EBV-CTLs from both an unrelated TPD and the respective SCD (no. 24/36). For 8 patients receiving EBV-CTLs from an unrelated TPD, T cell monitoring was performed, and EBV-specific T cells were found in 6 of them (75%, expansion performed for 1 of 2, total 88%; Figure 6B). Monitoring of T cells from 3 patients receiving SCD-originated EBV-CTLs showed that in one of them, EBV-specific T cells were detectable (33%, 2 of 2 after expansion, total 100%). For 6 of the 9 patients receiving EBV-CTLs from a related TPD, T cell monitoring was performed. By direct IFN-γ ELISpot assay, EBV-specific T cells were detected in 5 of them (83%, no expansion performed). Thus, irrespective of the donor type, functional EBV-specific T cells were detectable in the majority of patients after transfer of EBV-CTLs.

## Discussion

In this case series we describe the manufacture of 40 individualized EBV-specific T cell products isolated by magnetic separation after peptide pool stimulation and IFN-γ secretion, and their transfer into 37 patients with EBV-associated diseases. EBV-CTL products were generated from either SCDs (*n* = 13) or TPDs (*n* = 27) individually for each patient within 5 days (median; range 1–159 days) after donor identification. Prolonged intervals were mainly due to individual pretreatment regimens to reduce tumor burden. Among HSCT patients, the majority demonstrated a clinical response, with an SD of at least 70% (TPDs) and 90% (SCDs) of patients. While the CR rate was higher in patients receiving EBV-CTLs from SCDs (90%) as compared with TPDs (60%), there was no significant difference in the virologic response rate, with 73% clearing EBV from the peripheral blood in the TPD group and 60% in the SCD group. Few patients in both groups demonstrated reoccurrence or worsening of preexisting GvHD, while induction of de novo GvHD was observed exclusively in 2 patients of the SCD group, most likely unrelated to EBV-CTL transfer (°I skin plus °II intestine GvHD; °III liver, skin, and oral mucosa GvHD — one patient had a reduction in immunosuppression directly before the occurrence of GvHD, and the other received DLI and pembrolizumab in parallel with EBV-CTLs). No GvHD induction was observed in patients receiving EBV-CTL products either for EBV-associated PTLD after SOT or for EBV complications in immunodeficiency. In this group all patients received TPD EBV-CTLs, and 70% of patients showed partial or complete remission. EBV-related disease relapsed in one patient, who subsequently died of lymphoproliferation.

The series in the present study is, to our knowledge, the largest patient cohort reported to date to receive EBV-CTLs manufactured by using an IFN-γ cytokine secretion approach. Previously, Moosmann et al. pioneered this approach by manufacturing EBV-CTLs against known peptide antigens for 6 post-HSCT patients, 2 of whom benefited during early-stage PTLD and demonstrated sustained EBV-specific T cell expansion after adoptive transfer (36). Subsequently, Icheva reported this approach in 10 patients with EBV-associated complications after HSCT, with all EBV-CTLs manufactured from an SCD, and EBNA-1 protein or overlapping peptides as the sole target antigen (30). Seven patients showed clinical and/or virologic response. In the present case series, we extended this approach by (i) including multiple EBV antigens for several HLA types by introducing the EBV_Select peptide pool, (ii) including patients for whom the SCD was not available for T cell donation, and (iii) administering multiple infusions of EBV-specific CTLs if needed.

We demonstrated the feasibility of donor identification and EBV-CTL manufacture in a timely manner for most patients in need. From the alloCELL registry, we could provide suitable T cell donors for patients with EBV-seronegative SCDs or SCDs unavailable for T cell donation (37–39). Previous studies of ex vivo expanded EBV-CTLs transferred to partially HLA-matched patients with EBV-associated diseases have demonstrated efficacy in the majority of patients (25–29). Our approach provides a potential alternative to these previously published strategies of using banked expanded VSTs for rapid use on a best-HLA-match basis without the need for long-term in vitro expansion or manipulation. So far, no direct comparison of these 2 approaches has been conducted to our knowledge, but outcome and side effects observed in our cohort appear comparable to data from adoptive transfer of expanded EBV-CTLs.

Although this cohort reports the results of consecutive EBV-CTL manufacturing and adoptive transfer, it is a heterogeneous case series without the power of a prospective clinical trial. Despite this limitation, we attempted to address clinical and virologic efficacy by retrospective chart review. EBV-CTL transfer led to CR in 20 of 29 patients who were evaluated for clinical response. At the time of writing, 18 patients were alive and 12 patients had died, with 4 of the deaths related to EBV (2 in the HSCT/TPD group and 1 each in the HSCT/SCD and no-transplant/EBV-lymphoma groups). Of the remaining deaths, 4 were due to other infections; 1 to chronic GvHD; 1 to primary malignant disease relapse; 1 to a second malignancy; and 1 to multiorgan failure not otherwise classified. Thus, both SCD- and TPD-derived EBV-CTLs led to disease remission in the majority of treated patients, with a higher CR rate in patients receiving SCD-derived EBV-CTL products. From these data, however, we cannot conclude whether EBV-CTLs manufactured from SCDs or TPDs were more potent; prospective trials are needed to address this question.

All HSCT patients had received and were refractory to rituximab therapy prior to EBV-CTL administration. Exact documentation of response to all prior treatments was, unfortunately, not available, a limitation of this retrospective series. Some patients in both donor groups (SCDs, *n* = 4; TPDs, *n* = 5) continued to receive antibody (rituximab, brentuximab), immunomodulatory (bortezomib, checkpoint blockade), or cytotoxic therapy in parallel to EBV-CTL treatment according to the treating physician’s discretion (details in Supplemental Table 1A). We are unable to dissect the effects of individual treatment components in this retrospective analysis; however, parallel treatments were equally applied in both donor groups. The decline in EBV load in patients responding to EBV-CTL therapy occurred within 10–40 days after administration of the EBV-CTLs (Supplemental Figure 1A). None of these “responders” died of PTLD. In contrast, persistence of EBV in peripheral blood was associated with treatment failure in 4 closely monitored patients (Supplemental Figure 1B), 3 of whom ultimately died of PTLD. Although the study is limited by incomplete information for some patients and differences in local EBV-PCR assay techniques, these data support a close correlation between administration of EBV-CTLs, decline in EBV load, and control of PTLD.

No infusion-related side effects were reported in our patient cohort. Similarly, no graft failure (HSCT) or rejection (SOT) in the context of EBV-CTL administration was observed. Induction or aggravation of GvHD is a major concern when allogeneic T cells are administered, especially since immunosuppressive treatment is usually reduced to a minimum at the onset of refractory viral infections. Only 3 patients developed de novo GvHD following adoptive transfer of EBV-CTLs, 2 with mild 1 with severe GvHD. This case was reviewed by the treating physician, who associated the development of GvHD not to the transfer of EBV-CTLs but rather to the parallel administration of DLI. All 3 patients had undergone HSCT before EBV-CTL transfer, and in all cases the SCD served as EBV-CTL donor. The transferred EBV-CTL cell count in all 3 patients with new GvHD was close to the median of the EBV-CTL cell count of all patients; thus, there was no relationship between cell dose and occurrence of GvHD. It may be speculated that engraftment of SCD-derived CTLs was better than that of TPD-derived CTLs; however, the numbers are too small and patient groups too heterogeneous to fully address this idea in the present retrospective patient evaluation. Two HSCT patients in the TPD group had no GvHD before CTL transfer; none of them developed de novo GvHD after EBV-CTL transfer. In this group, GvHD symptoms became aggravated in 3 of 9 patients with preexisting GvHD — in 2 of them attributable to either checkpoint inhibitor (*n* = 1) or sorafenib (*n* = 1) treatment. Further, GvHD could not be clearly attributed to transfer of EBV-specific T cells in any of the patients who developed GvHD.

Monitoring of EBV-specific T cells after adoptive transfer was performed in a subset of patients, and most patients had detectable EBV-specific T cells by IFN-γ ELISpot analysis. The numbers are too small and time points of monitoring to diverse to allow correlation of the magnitude of T cell responses and clinical or virologic outcome. EBV-specific T cell responses detected shortly after EBV-CTL infusion might be indicative of the development of endogenous T cell responses rather than a direct effect of EBV-CTL transfer. The question of whether the transferred EBV-CTLs directly led to the therapeutic effect or whether this was mediated by induction of endogenous T cell responses remains open. In a number of case reports, it was shown that in patients lacking VSTs, adoptive transfer of VSTs isolated using the cytokine capture system resulted in detectable VSTs (31, 40, 41). Further, in a cohort of pediatric solid organ graft recipients, an increase in EBV-specific T cells upon reduction of immunosuppression and treatment with rituximab was observed, indicating that the endogenous immune responses can be boosted by release of viral antigens due to rituximab-mediated cell lysis (42), which might apply to the mechanism of adoptively transferred VSTs as well. We recently reported that EBV antigens released from EBV-transformed B lymphoblastoid cell lines promote EBV-specific memory T cell responses (43). Furthermore, we have previously shown by T cell receptor (TCR) sequencing in a patient receiving TPD-derived EBV-CTLs that both persistence and expansion of transferred cells — as well as induction of endogenous EBV-specific T cell responses, thereby broadening the antigenic repertoire — can occur (31). Single-cell sequencing studies are starting to elucidate TCR sequences that confer therapeutic efficacy and protective anti-EBV immunity (44). Future studies, including immune monitoring prior to EBV-CTL infusion as well as discrimination between donor- and recipient-derived T cells after transfer, are required to elucidate the mechanism as well as the therapeutic efficacy of adoptive T cell transfer. More broadly, TCR transfer in autologous T cells may be an alternative though elaborate option for patients lacking a suitable T cell donor.

In conclusion, our data support the notion that adoptive transfer of EBV-CTLs from SCDs and related or unrelated TPDs enriched by the CliniMACS CCS IFN-gamma process is feasible, clinically effective, and safe. Using patient-specific EBV-CTLs manufactured via direct magnetic isolation circumvents the need for prolonged in vitro expansion and good manufacturing practice–compliant (GMP-compliant) banking of EBV-CTL products despite rapid availability. This treatment seems promising for immunocompromised patients with refractory EBV-associated diseases even beyond HSCT. Limited side effects and low organ toxicity make this approach attractive for patients with preexisting organ dysfunction. However, prospective clinical trials are required to address questions regarding best available donor, best manufacturing strategy, and optimal cell dose and dosing intervals, as well as the mode of action and persistence of the transferred T cells.

## Methods

### T cell donor registry (alloCELL).

The allogeneic T cell donor registry (alloCELL) at the Institute of Transfusion Medicine and Transplant Engineering (Hannover Medical School) currently records more than 3,500 HLA-typed donors with known VST frequencies against common human viruses. Following receipt of written informed consent, antiviral T cell frequencies were determined by IFN-γ ELISpot assay (see “Patient follow-up”) using residual blood samples originating from platelet apheresis disposable kits used for routine platelet collection from regular healthy blood donors of the Institute of Transfusion Medicine and Transplant Engineering.

### Donor pretesting.

Donor EBV serostatus was determined by analysis of anti-EBV IgG antibodies in serum samples using a line immunoassay (recomLine, Mikrogen Diagnostik). The IFN-gamma cytokine secretion assay (CSA, Miltenyi Biotec), which is largely analogous to the clinical-grade manufacturing procedure, was performed to determine EBV-specific T cell frequencies in selected donors and to predict the expected efficiency in the manufacturing process (35). PBMCs were isolated by density gradient centrifugation and seeded into 24-well cell culture plates with 1 × 10^7^ cells per well in TexMACS medium (Miltenyi Biotec). Following an overnight resting period, cells were stimulated with PepTivator EBV_EBNA-1 alone or in combination with PepTivator EBV_Consensus (both from Miltenyi Biotec) for 4 hours. As NC, cells were kept unstimulated. CSA was performed according to manufacturer’s instructions. Activated IFN-γ–producing T cells were captured during the magnetic cell enrichment process using anti–IFN-γ–phycoerythrin (anti–IFN-γ–PE) antibodies and paramagnetic anti-PE microbeads. Aliquots of the respective cell fractions collected before and after enrichment were used for analysis of IFN-γ^+^ T cell subsets by multicolor flow cytometry using the following antibodies: anti-CD3–FITC (SK7), anti-CD4–Alexa Fluor700 (RPA-T4), anti-CD8–allophycocyanin (anti-CD8–APC, SK1), and anti-CD45–APC-H7 (2D1, all BD Biosciences); and anti–IFN-γ–PE (Miltenyi Biotec). For discrimination of alive and dead cells, samples were incubated with 7-aminoactinomycin D (7-AAD, BD Biosciences) just prior to analysis. Samples were acquired with a 10-color BD FACSCanto (BD Biosciences) and analyzed using BD FACSDiva software (version 8.0.1, BD Biosciences).

### Generation of clinical-grade EBV-CTL products.

Donor leukapheresis products were enriched for IFN-γ–secreting cells in compliance with European Union GMP starting with 1 × 10^9^ leukocytes in response to MACS GMP PepTivators EBV_EBNA-1 and EBV_Select (GMP-grade product consisting of the same peptides as EBV_Consensus) using CliniMACS CCS IFN-γ and CliniMACS Plus or Prodigy device (all Miltenyi Biotec). The enrichment process was performed according to the manufacturer’s instructions for both devices (35, 37). Aliquots of the leukaphereses and in-process samples (preenrichment, final product, negative fraction) were taken for quality control using flow cytometry (35). All products (*n* = 40) fulfilled the specification criteria. The final products were divided into portions according to the dosage. For cryopreservation, products were supplemented with 7.5% DMSO, processed in a controlled-rate freezer, and finally transferred to the vapor phase above liquid nitrogen for storage. Moreover, leukaphereses and final products were tested for sterility by using a fully automated microbial detection system. Aliquot samples of cryopreserved T cell products were subjected to quality control as described in ref. 35.

### Clinical data collection and response criteria.

Clinical data were collected by standardized questionnaire. Follow-up ranged from 4 weeks to 77 months (median 34.5 months) after EBV-CTL transfer. Data collected included reason for transfer, local histology report, numbers of EBV-CTL transfers, GvHD status before and after transfer, virologic response, and clinical response. Response data were collected for all patients who had a follow-up of at least 3 weeks after the first CTL transfer. CR was defined as disappearance of all lesions on imaging if present before treatment and resolution of PTLD-related symptoms. PR was defined as at least a 25% reduction in tumor volume and no appearance of new lesions. SD was defined as no change in tumor volume greater than 25%.

EBV-PCR was carried out according to the respective local laboratory standards but was consistent within individual patients. Complete virologic response was defined as disappearance of EBV load according to PCR. Partial virologic response was defined as viral load reduced by at least 1 log_10_ but still measurable. All other situations were defined as virologic nonresponse.

### Monitoring of EBV-specific T cell responses after EBV-CTL transfer.

For determination of EBV-specific T cell frequencies in patient blood, the IFN-γ ELISpot Assay was performed as described previously (45). Briefly, PBMCs isolated from patient blood by density centrifugation were allowed to rest overnight in RPMI (Lonza) supplemented with 10% human AB serum (c.c.pro). Rested PBMCs were cultured in anti–IFN-γ–precoated ELISpot plates (Lophius Biosciences) for 16–18 hours at a density of 2.5 × 10^5^ or 5.0 × 10^5^ cells/well and stimulated with PepTivator EBV_EBNA-1 or EBV_Consensus (both 1 μg/mL per peptide; Miltenyi Biotec). Unstimulated cells served as NC, and cells supplemented with 1 μg/mL staphylococcal enterotoxin B (SEB, MilliporeSigma) served as positive control. Following overnight incubation, IFN-γ secretion was detected using an AID iSpot Reader System and AID ELISpot software version 8.0 (Autoimmun Diagnostika). IFN-γ–positive cells were counted and expressed as the number of spots per well. The mean number of spots in the NC was subtracted from the mean number of spots in the antigen wells.

To determine levels of low-frequency EBV-specific T cells after adoptive T cell transfer, we cultured isolated PBMCs in the presence of PepTivator EBV_EBNA-1 or EBV_Consensus (both 1 μg/mL per peptide; Miltenyi Biotec) for 7 days in the presence of 50 IU/mL IL-2 (Peprotech). Subsequent to this expansion period, cells were harvested and subjected to ELISpot assay as described above.

### Data analysis.

Data were analyzed by Microsoft Excel 2010 (Microsoft). Summarizing graphs were generated using GraphPad Prism 8.2.2 (GraphPad Software). For display of flow cytometric data, FlowJo v10 (FlowJo LLC, BD Biosciences) was used.

### Statistics.

Descriptive statistics were used to determine median, mean, and range data. Statistical significance was calculated using Kruskal-Wallis test, followed by Dunn’s multiple-comparisons test. A *P* value less than 0.05 was considered significant.

### Study approval.

Written informed consent was obtained from donors in the allogeneic T cell registry (alloCELL), established at the Institute of Transfusion Medicine and Transplant Engineering, Hannover Medical School (ethics committee votes 3331-2016, 3639-2017). Clinical data collection was approved by the Institutional Review Board of Hannover Medical School (ethics committee vote 3207-2016).

## Author contributions

BEV and BMK designed and supervised the program. AB and STZ generated and evaluated the donor selection, manufacturing, and immune-monitoring data. BL collected and analyzed the clinical data. AB was mainly responsible for manuscript writing and data analysis with respect to TPDs and patient monitoring. BL was mainly responsible for data analysis with respect to clinical outcome. STZ was mainly responsible for data analysis with respect to SCDs. Co–first authorship was assigned in alphabetical order. RSF, RB, GB, GC, BF, JG, LH, JH, MH, PH, EJ, KK, OK, NK, SM, RM, MN, MP, HCR, ES, MS, CS, RS, NKS, RUT, MV, and DW treated patients and provided clinical data. LG, HGH, LA, and RB manufactured EBV-CTLs. SR participated in immunological analysis. AB, STZ, BL, BEV, and BMK wrote the manuscript. All authors read and approved the final version of the manuscript.

## Supplementary Material

Supplemental data

ICMJE disclosure forms

## Figures and Tables

**Table 5:**
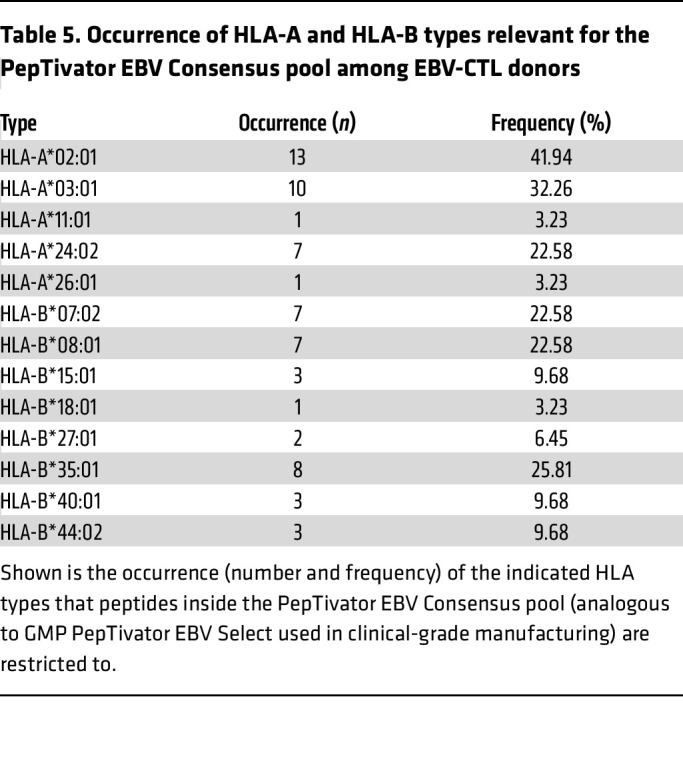
Occurrence of HLA-A and HLA-B types relevant for the PepTivator EBV Consensus pool among EBV-CTL donors

**Table 4:**
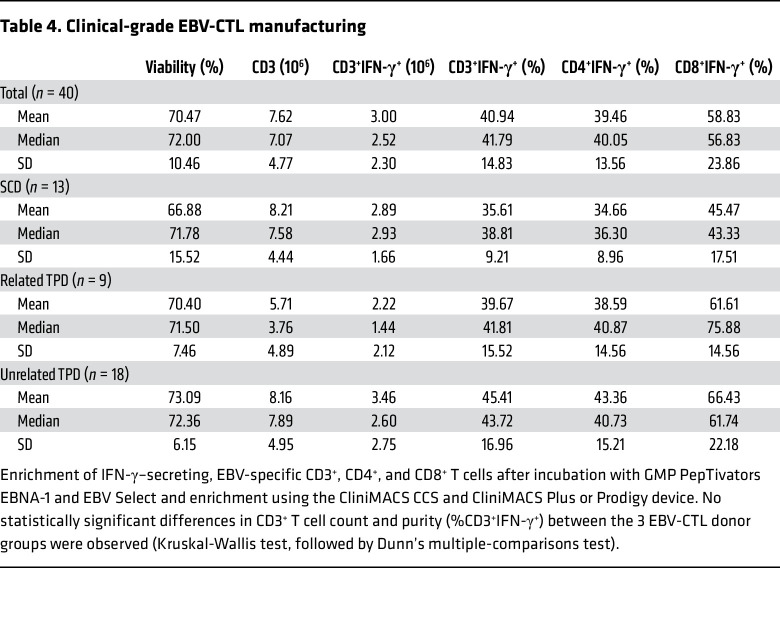
Clinical-grade EBV-CTL manufacturing

**Table 2:**
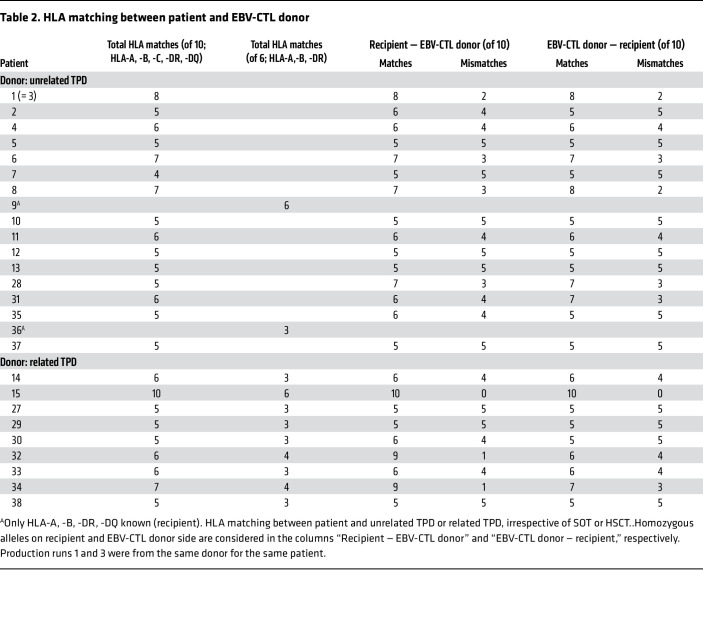
HLA matching between patient and EBV-CTL donor

**Table 1:**
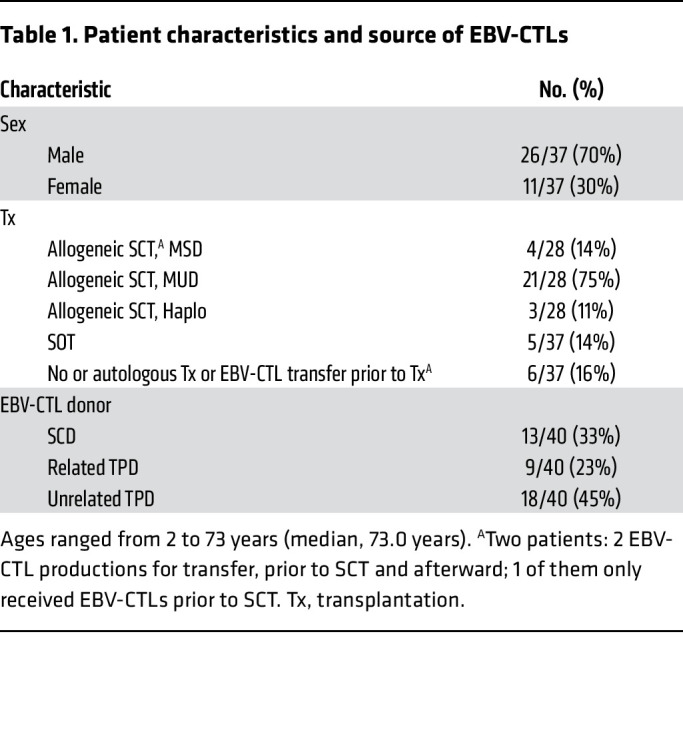
Patient characteristics and source of EBV-CTLs

**Table 3:**
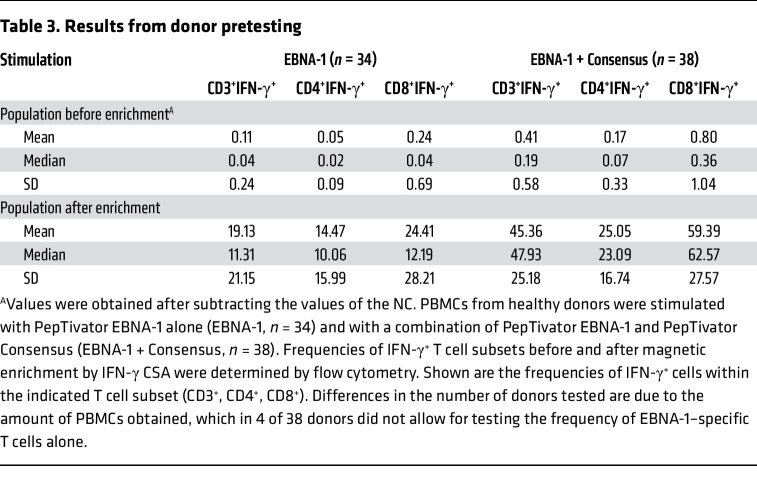
Results from donor pretesting

**Table 6:**
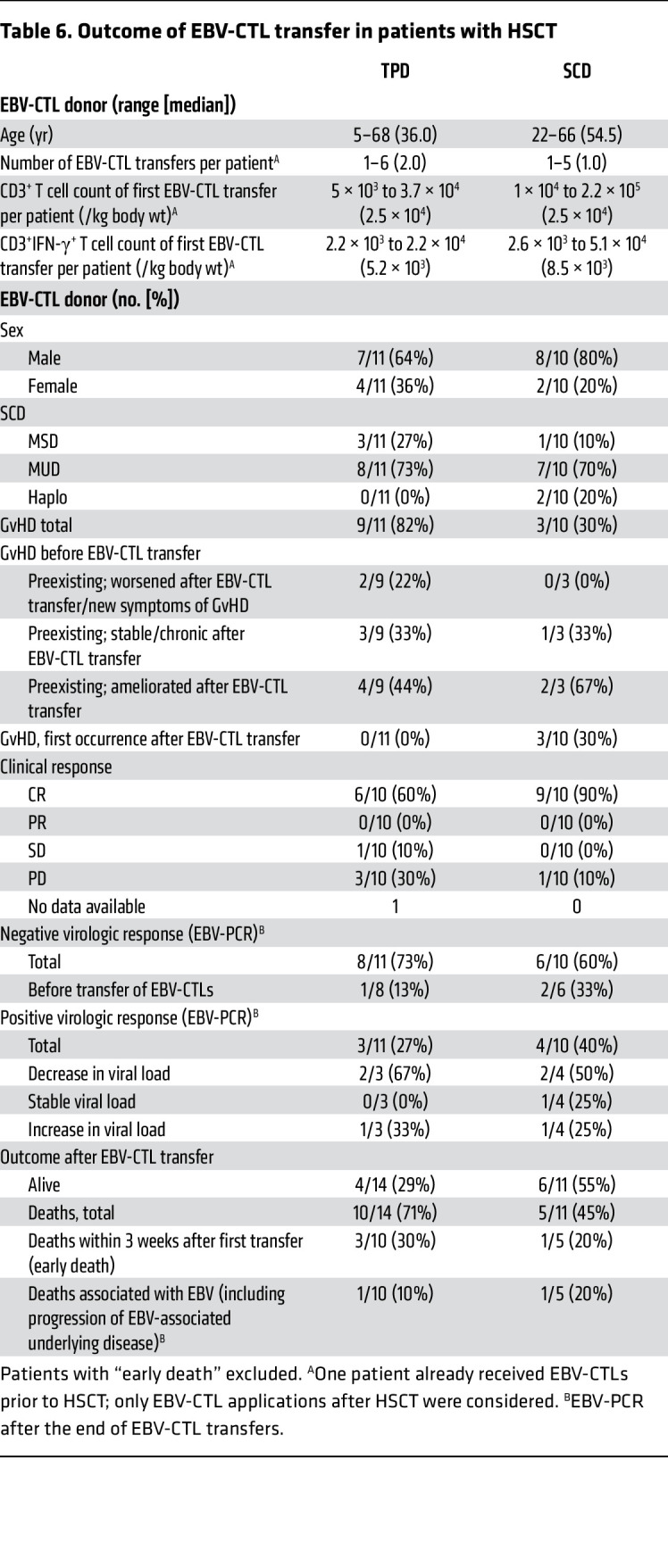
Outcome of EBV-CTL transfer in patients with HSCT

## Version 1 05/09/2023

In-Press Preview

## Version 2 06/15/2023

Electronic publication
