# Supplementary material for: Patient-tailored adoptive immunotherapy with EBV-specific T cells from related and unrelated donors
Source: J Clin Invest. 2023 Jun 15;133(12):e163548. doi: 10.1172/JCI163548 (PMC10266790; doi:10.1172/JCI163548)
Supplement: Supplemental data [file jci-133-163548-s073.pdf]

Supplemental Material

Patient-tailored adoptive immunotherapy with EBV-specific T cells from related and unrelated donors

A Bonifacius, et al.

Supplemental conflict-of-interest statement:

The following authors have declared a potential conflict of interest: GC, honoraria (Novartis, Servier), travel support (Jazz Pharmaceuticals), data safety monitoring advisory board (Jazz Pharmaceuticals); JH, honoraria (AbbVie, Amgen, Bristol-Myers Squibb, Celgene, Deutscher Ärzte-Verlag, Georg Thieme Verlag, Gilead Sciences, Janssen-Cilag, Jazz Pharmaceuticals, MedKom Akademie, MSD Sharp & Dohme, Neovii, NewConceptOncology, NIO Niedersachsen, Novartis, Mundipharma, Pfizer); HGH, leadership or fiduciary role (State and Communal Blood Transfusion Services Working Party); EJ, honoraria (JAZZ: AML), travel support (Neovii, Gilead Sciences); NK, leadership or fiduciary role (EBMT president); BMK, leadership or fiduciary role (German Pediatric PTLD study group); HCR, grants or contracts (AstraZeneca, Gilead Sciences), consulting (Vertex, Roche), honoraria (AbbVie, AstraZeneca, Vertex, Merck), travel support (AbbVie), patents (nos. 20090010927, 20214527.2-1112), cofounder (CDL Therapeutics); RUT, grants or contracts (Atara Biotherapeutics, Roche), consulting (Atara Biotherapeutics), honoraria (Atara Biotherapeutics, Roche), expert testimony (Atara Biotherapeutics), travel support (Atara Biotherapeutics, Janssen-Cilag, Roche, BeiGene, AbbVie), leadership or fiduciary role (German PTLD study group, German Society of Hematology and Oncology); DW, grants or contracts (Novartis), consulting (Sanofi, Incyte), honoraria (Mallinckrodt, Takeda), travel support (Gilead Sciences), data safety monitoring advisory board (Novartis, Behring).

Supplemental Figure 1

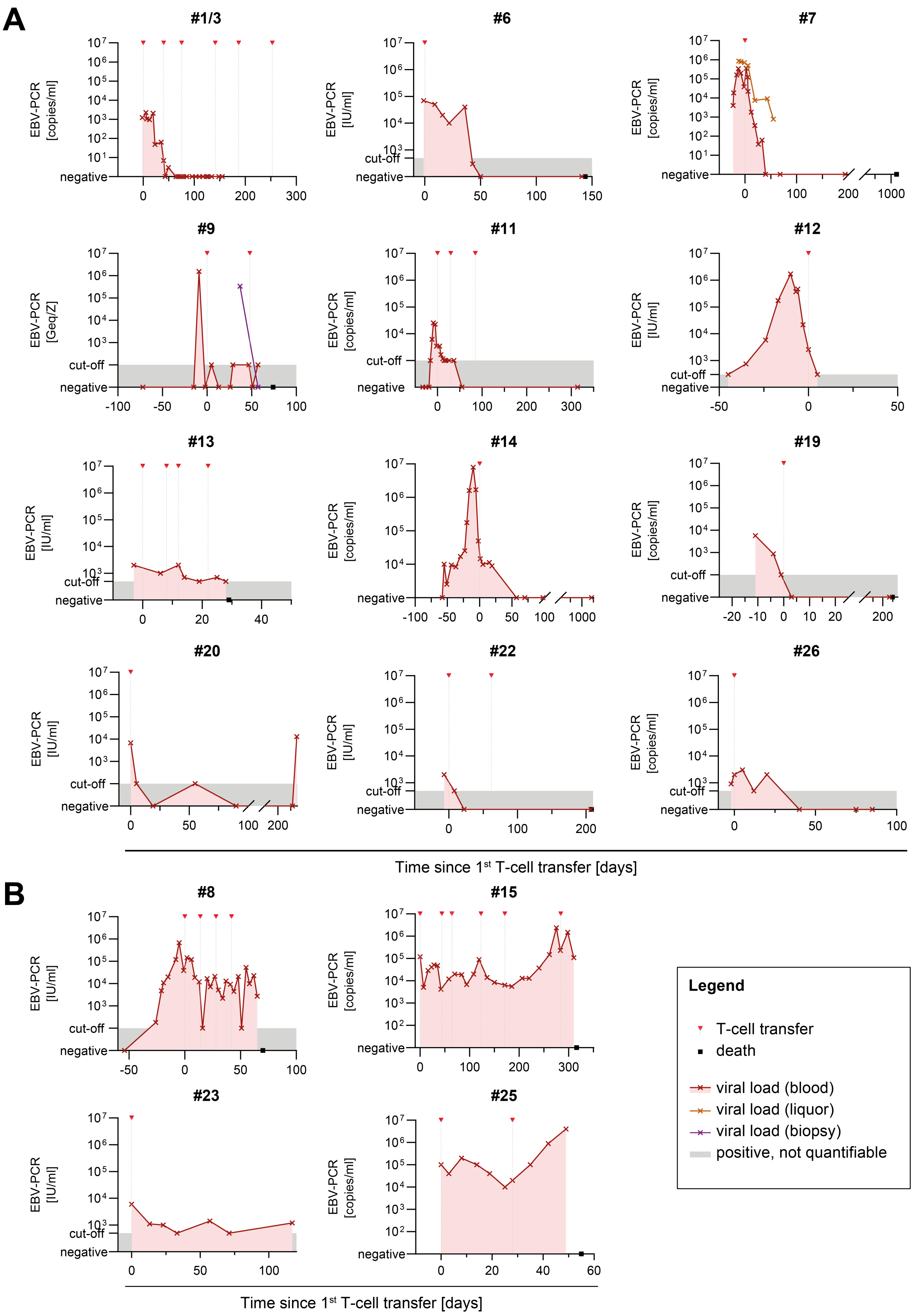

Supplemental Figure 1: Viral load in patients receiving EBV-CTLs.
(A) Patients with reduction in viral load ("responders"). **(B)** Patients with persisting viral load ("non-responders").

Patient-tailored adoptive immunotherapy with EBV-specific T cells from related and unrelated donors

Supplemental Table 1A

	No.	age at 1st transfer	Gender	SC donor / organ	underlying disease	reason for EBV-CTL transfer	histologic features of PTLD	previous therapy of EBV/PTLD	change in immunosuppression	anti-EBV/PTLD therapy in parallel to or after EBV-CTL transfer	EBV-CTL donor	Search - donor identification (days)	Donor identification - Manufacturing (days)	CD3+ cell count of 1st transfer (per kg BW)	total CD3+ cell counts of all transfers (per kg BW)	purity (% CD3+ IFN-γ+ T cells)	number of transfers
HSCT patient; EBV-CTL from SCD (Ia)	16	22	m	MUD	relapsed AML	PTLD	Hodgkin-like	rit	no	none	SCD	-	40	2.50E+04	2.50E+04	25.0	1
	17	54	m	MSD	multiple myeloma	PTLD	no biopsy	n.a.	n.a.	n.a.	SCD	-	-	3.04E+04	3.04E+04	17.7	1
	18	73	m	MUD	MDS	PTLD	DLBCL	rit, ster, ofatumumab	no	none	SCD	-	2	2.22E+05	2.22E+05	38.8	1
	19	66	f	MUD	AML	PTLD	classical Hodgkin's disease	rit, CHOP	stop of MMF and calcineurin inhibitor	none	SCD	-	2	1.21E+05	1.21E+05	42.1	1
	20	65	m	MUD	multiple myeloma	PTLD	no biopsy	rit	everolimus instead of calcineurin inhibitor	none	SCD	-	5	2.50E+04	2.50E+04	43.3	1
	21	22	m	haplo	AML	PTLD	polymorphic PTLD	rit	n.a.	rit	SCD	-	14	2.50E+04	5.00E+04	29.4	2
	22	59	f	MUD	MDS	PTLD	CD30 and EBV positive PTLD	rit	no	none	SCD	-	7	5.00E+04	7.07E+04	42.1	2
	23	55	m	MUD	NKT-NHL (EBV positive)	progression of EBV-positive NKT-NHL	-	-	stop of immunosuppression	pembrolizumab	SCD	-	-	5.00E+04	5.00E+04	40.5	1
	24	28	f	MUD	EBV-associated lymphoproliferation	EBV-associated lymphoproliferation, severe/uncontrolled EBV-infection, EBV encephalitis	-	ganciclovir, aciclovir, bort, rit	taper and stop of everolimus	rit	SCD	-	-	1.00E+04	1.40E+05	25.8	5 (14)
	25	44	m	haplo	aplastic anemia + PNH	PTLD	non-destructive PTLD, plasmacytic hyperplasia	rit, 1x EBV-CTL	no	none	SCD	-	4	2.50E+04	2.50E+04	34.1	1
	26	62	m	MUD	MDS-MPN overlap	PTLD	DLBCL	rit	yes, details n.a.	mtx + rit (OPTIMAL protocol)	SCD	-	-	2.50E+04	5.00E+04	33.4	2
HSCT patient; EBV-CTL from TPD (Ib)	1	52	m	MUD	AML/MDS	PTLD	DLBCL	rit, R-CHOP, bren	no	bren, IVIG	unrel TPD	0	108	1.00E+04	4.24E+04	47.2	3 (6)
	2	58	m	MUD	multiple myeloma	EBV re-activation with necrotisizing encephalitis	-	rit	taper and withdrawal of CsA	none	unrel TPD	0	6	1.00E+04	1.09E+04	29.0	2
	3	52	m	MUD	AML/MDS	PTLD	DLBCL	rit, R-CHOP, bren	no	bren, IVIG	unrel TPD	see #1	see #1	1.00E+04	4.24E+04	46.1	3 (6)
	4	68	m	MUD	myelofibrosis	EBV re-activation	-	rit	no	none	unrel TPD	0	12	2.50E+04	2.50E+04	38.6	1
	5	28	f	MUD	AML	PTLD	no biopsy	rit, ster	no	none	unrel TPD	0	1	2.50E+04	2.50E+04	72.8	1
	6	50	f	MUD	AML	EBV re-activation	-	rit	taper and withdrawal of CsA and MMF	none	unrel TPD	3	10	2.51E+04	2.50E+04	72.2	1
	7	12	m	MUD	CMMRD, relapsed T-NHL	PTLD	DLBCL	rit i.v. + i.th., ganciclovir, cpm	taper of ster	ganciclovir	unrel TPD	6	4	5.06E+03	5.00E+03	43.9	1
	8	14	f	MUD	secondary MDS after Ewing sarkoma	PTLD	DLBCL	rit	taper of CsA	bren	unrel TPD	10 (search - manufacturing)		1.00E+04	4.00E+04	30.0	4
	9	65	m	MSD	multiple myeloma	PTLD	monomorphic PTLD	rit, R-CHOP + bort	no	rit, R-CHOP	unrel TPD	2	4	2.50E+04	5.00E+04	76.8	2
	10	38	m	MUD	relapsed AML	PTLD	B-NHL with plasmacytic differentiation	rit	no	rit	unrel TPD	15	1	2.50E+04	2.50E+04	41.8	1
	11	5	f	MSD	familial HLH	PTLD	DLBCL	rit, foscavir	stop of tac	rit, foscavir, bren	unrel TPD	2	4	2.50E+04	7.50E+04	46.0	3
	12	14	f	MUD	relapsed ALCL	EBV re-activation	-	rit	taper and withdrawal of CsA	none	unrel TPD	4	1	1.00E+04	1.00E+04	39.3	1
	13	40	m	MUD	ALL	PTLD	highly aggressive B-cell lymphoma	rit	no	none	unrel TPD	2	6	1.00E+04	4.00E+04	31.3	4
	14	17	m	MUD	VSAA	PTLD	polymorphic PTLD	rit	sirolimus instead of tac	rit	rel TPD	-	4	2.50E+04	2.50E+04	20.8	1
	15	36	m	MSD	AML	PTLD	polymorphic PTLD	rit, vcr	stop ofCsA	bort, bren, nivulomab	rel TPD	-	5	3.69E+04	1.60E+05	58.9	6
SOT patient; EBV-CTL from TPD (II)	27	12	m	kidney	nephronophtisis	PTLD	no biopsy	rit, mCOMP	no	rit, bren	rel TPD	-	39	4.17E+04	4.17E+04	22.5	1
	28	10	m	liver	Alagille syndrome	PTLD	monomorphic PTLD with features of DLBCL	rit, intrathecal therapy (rit, cyarabine, mtx, prednisone)	stop of tac and MMF	none	unrel TPD	3	67	2.50E+04	1.25E+05	49.9	5
	29	14	f	heart	dilatative cardiomyopathy	PTLD	DLBCL	rit, mCOMP	everolimus instead of MMF, taper of CsA	none	rel TPD	-	53	1.00E+04	3.00E+04	41.8	3
	30	52	m	liver	alcoholic cirrhosis	PTLD	plasmablastic lymphoma / monomorphic PTLD	VIP prephase, CHOP	stop of ster, taper of tac	none	rel TPD	-	3	2.50E+04	5.00E+04	20.3	2
	31	63	f	kidney	glomerulonephritis	PTLD	small cell B-NHL with partially blastic, partially plasmacytic differentiation	rit, bort, IVIG, R-CHOP-21	taper of CsA	none	unrel TPD	6	20	2.51E+04	1.25E+05	43.1	4
no Tx; EBV-CTL from TPD (III)	32	47	m	-	EBV-associated Hodgkin lymphoma, EBV-associated lymphoproliferation	EBV-associated lymphoproliferation	-	R-ABVD, R-BEACOPPesk, R-AVD, rit, bren	..	none	rel TPD	-	25	1.00E+04	3.00E+04	51.8	3
	33	6	m	prior to SCT	immunodeficiency (X-MEN syndrome)	chronic EBV infection, EBV-induced hepatitis	-	rit	-	none	rel TPD	-	5	1.00E+04	3.50E+04	58.8	2
	34	32	m	prior to SCT	EBV-associated Hodgkin lymphoma, suspected immunodeficiency	high EBV viral load	-	rit, tocilizumab	-	rit, R-CHOP, bren, nivulomab	rel TPD	-	11	1.50E+04	4.34E+04	42.8	3
	35	23	m	-	EBV-associated Hodgkin lymphoma	EBV-associated refractory Hodgkin lymphoma	-	ABVD, R-ICE, bren-DHAP, irradiation, nivulomab, rit	-	bren, nivulomab	unrel TPD	58	1	2.49E+04	5.00E+04	58.4	2
	36	28	f	prior to SCT	EBV-associated lymphoproliferation	EBV-associated lymphoproliferation, severe/uncontrolled EBV-infection, EBV encephalitis	-	ganciclovir, aciclovir, bort, rit	-	none	unrel TPD	2	1	1.00E+04	1.40E+05	43.5	9 (14)
EBV-CTL not used	37	52	m	auto	EBV-associated Hodgkin lymphoma	EBV-associated lymphoproliferation	-	rit, cpm, ster	-	-	unrel TPD	0	5	n.a.	-	n.a.	0
	38	16	m	MUD	secondary AML after Ewing sarkoma	PTLD	no biopsy	rit	n.a.	-	rel TPD	-	5	2.51E+04	-	39.4	0
	39	2	f	haplo	immunodeficiency (CD27 deficiency)	preemptive therapy, finally not necessary	-	none	-	-	SCD	-	159	2.50E+04	-	39.1	0
	40	6	m	MUD	immunodeficiency (X-MEN syndrome)	chronic EBV infection, EBV-induced hepatitis	-	rit, allogenic SCT	-	-	SCD	-	26	9.55E+03	-	51.5	0

patients with two EBV-CTL manufacturing processes: No. 1 = No. 3; No. 24 = No. 36; No. 33 = No. 40

abbreviations: BW = body weight; rit = rituximab; bren = brentuximab-vedotin; ster = steroids; bort = bortezomib; cpm = cyclophosphamide; vcr = vincristin; mtx = methotrexate; CsA = cyclosporin A; tac = tacrolimus; MMF = mycophenolatmofetil

Patient-tailored adoptive immunotherapy with EBV-specific T cells from related and unrelated donors

Supplemental Table 1B

	No.	GvHD before EBV-CTL transfer	details of GvHD before EBV-CTL transfer	GvHD after EBV-CTL transfer	details of GvHD after EBV-CTL transfer	reason for GvHD after EBV-CTL transfer	transfer of unspecific DLI	clinical response	EBV-PCR (blood) after EBV-CTL transfer	follow-up (months)	patient alive/deceased	reason for death
HSCT patient; EBV-CTL from SCD (Ia)	16	yes	cerebral vasculitis (imaging)	none	-	-	no	CR	negative**	77	alive	-
	17	none	-	yes	skin 1°, bowel 2°	n.a.	n.a.	CR	positive but not quantifiable	31	alive	-
	18	yes	acute bowel 4°, chronic articulations 1°	none	-	-	no	early death (n.a.)			deceased	multiorgan failure, EBV encephalitis, septic shock, GvHD
	19	yes	skin 2°, bowel 1°	none	-	-	no	CR	negative	11	deceased	adenovirus-mengoencephalitis + bacterial sepsis
	20	none	-	yes	skin moderate, bowel mild	reduction of everolimus	no	CR	negative	51	alive	-
	21	none	-	none	-	-	no	CR	negative**	52	alive	-
	22	yes	bowel 1°	chronic	bowel chronic (limited disease, NIH-SS mild)	unknown (association with EBV possible)	no	CR	negative	6	deceased	adenovirus encephalitis, pneumonia, liver failure
	23	none	-	yes	skin 3°, oral mucosa 3°, liver 3°	association with DLI and pembrolizumab	yes	CR	positive (stable)	6	deceased	influenza, sepsis
	24	none	.	none	-	-	no	CR	positive but not quantifiable	44	alive	-
	25	none	-	none	-	-	no	PD	positive (stable)	1	deceased	progression of PTLD
	26	none	-	none	-	-	yes	CR	negative	40	alive	-
HSCT patient; EBV-CTL from TPD (Ib)	1	yes	skin 3°; resolved before EBV-CTL transfer	none	-	-	no	CR	negative	71	alive	-
	2	none	-	(none)	-	-	no	early death (n.a.)			deceased	progression of EBV-associated necrotizing encephalitis
	3	yes	skin 3°; resolved before EBV-CTL transfer	none	-	-	no	CR	negative	71	alive	-
	4	yes	skin 2°, liver 1°, bowel 2°, oral mucosa 2°	yes, chronic	skin 2°, liver 1°, bowel 2°, eyes 1°	unknown (association with EBV possible)	no***	n.a.	negative**	3	deceased	chronic GvHD
	5	yes	skin 2°	(none)	-	-	no	early death (n.a.)			deceased	multiorgan failure (presumably EBV-associated)
	6	yes	bowel 1°	yes, new symptoms	skin 3°	association with DLI (and sorafenib)	yes	CR	negative	5	deceased	progression of AML
	7	yes	skin 2°, bowel 3°	no new symptoms	no new symptoms	-	no	CR	negative (liquor: low positive)	38	deceased	secondary malignoma (glioblastoma multiforme)
	8	none	-	none	-	-	no	PD	positive (decline)	2	deceased	progression of PTLD
	9	yes	skin 2°	none	-	-	no	PD	negative	2	deceased	multiorgan failure
	10	yes	skin 3°, bowel 3°	(none)	-	-	no	early death (n.a.)			deceased	progression of PTLD and AML
	11	none	-	none	-	-	no	CR	negative	11	alive	-
	12	yes	skin 1°	none	-	-	no	CR	negative	42	alive	-
	13	yes	skin chronic (limited diesease, NIH-SS moderate)	none	-	-	no	SD	positive (decline)	1	deceased	atypic pneumonia
	14	yes	skin 3°	no new symptoms	-	-	no	CR	negative	48	alive	-
	15	yes	skin 2°, bowel 1°	yes, new symptoms	skin 4°, bowel 2°, liver 3°	association with nivulomab	no	PR, finally PD	positive (increase)	10	deceased	progression of PTLD, severe GvHD following nivulomab, septic shock
SOT patient; EBV-CTL from TPD (II)	27	none	-	none	-	-	-	SD****	negative	69	alive	-
	28	none	-	none	-	-	-	CR before transfer of EBV-CTL	negative**	77	alive	-
	29	none	-	none	-	-	-	CR before transfer of EBV-CTL	negative**	61	alive	-
	30	none	-	none	-	-	-	CR	negative**	58	alive	-
	31	none	-	none	-	-	-	CR	positive (decline)	27	alive	-
no Tx; EBV-CTL from TPD (III)	32	-	-	none	-	-	-	PD	positive (decline)	3	deceased	progression of EBV-associated lymphoproliferation
	33	-	-	none	-	-	-	SD	negative after SCT*	64	alive	-
	34	-	-	none	-	-	-	CR after SCT	negative after SCT	22	alive	-
	35	-	-	none	-	-	-	PR****	negative**	40	alive	-
	36	-	-	none	-	-	-	CR	positive (decline)	44	alive	-
EBV-CTL not used	37	-	-	-	-	-	-	-	-	-	deceased	multiorgan failure, uncontrolled EBV reactivation and lymphoproliferation
	38	-	-	-	-	-	-	-	-	-	deceased	aspergillosis, progression of PTLD
	39	-	-	-	-	-	-	-	-	-	alive	-
	40	-	-	-	-	-	-	-	-	-	alive	-

patients with two EBV-CTL manufacturing processes: No. 1 = No. 3; No. 24 = No. 36; No. 33 = No. 40
*patient No. 33: increasing EBV load despite EBV-CTL transfer, CR after HSCT without further EBV treatment
** EBV-PCR negative before transfer of EBV-CTL
***patient No. 4: no DLI but transfer of CMV-CTL 3 months before transfer of EBV-CTL
****patient No. 27 and 35: clinical response SD/PR but complete metabolic response (PET)

Patient-tailored adoptive immunotherapy with EBV-specific T cells from related and unrelated donors

Supplemental Table 2: Clinical-grade EBV-CTL manufacturing, CliniMACS Plus vs. CliniMACS Prodigy.

Enrichment of IFN γ-secreting, EBV-specific $CD3^+$, $CD4^+$, and $CD8^+$ T cells after stimulation with GMP-grade PepTivators EBV_EBNA-1 and EBV_Select and enrichment using the CliniMACS CCS and CliniMACS Plus or Prodigy device.

		Viability [%]	$CD3^+$ [10^6]	$CD3^+$/IFN-γ^+ [10^6]	$CD3^+$/IFN-γ^+ [%]	$CD4^+$/IFN-γ^+ [%]	$CD8^+$/IFN-γ^+ [%]
Plus (n=13)	Mean	68.1	3.95	1.55	36.84	36.03	64.48
	Median	71.07	2.44	0.93	41.81	39.36	80.18
	SD	7.1	3.63	1.52	15.18	15.15	27.65
Prodigy (n=27)	Mean	71.6	9.39	3.7	42.91	41.11	55.67
	Median	72.94	9.53	3.16	41.76	40.1	55.11
	SD	11.7	4.24	2.31	14.53	12.7	21.79
